# Supplementary material for: Describing, predicting and explaining adherence to total skin self-examination (TSSE) in people with melanoma: a 12-month longitudinal study
Source: BMJ Open. 2022 Aug 30;12(8):e056755. doi: 10.1136/bmjopen-2021-056755 (PMC9438032; doi:10.1136/bmjopen-2021-056755)
Supplement: Supplementary data [file bmjopen-2021-056755supp001.pdf]

Supplementary File S1

Multivariate analyses

The main analyses reported in the manuscript are univariate logistic regressions. As a supplementary analysis, any predictors that were significant at p<.1 were entered into further multivariate analyses. These multivariate analyses are shown below in Tables S1 and S2.

When intention, anxiety and depression (i.e. all predictors where p<.1) were included in the analysis of non-adherent versus adherent participants, intentions about TSSE and depression remained significant, each explaining unique variance. People who were non-adherent were more depressed and more likely to intend to conduct TSSE more frequently than recommended than people who were adherent.

When action planning and self-efficacy (as predictors where p<.1) were included in a multivariate analysis of the drop offs versus the adherent participants, neither was significant. This may reflect the fact that efficacy is a theoretical precursor of action planning, and as such, the two will be highly inter-related and may explain the same variance.

Table S1: Multivariate logistic regression analyses of demographic, cognitive and emotional factors in people who are **non-adherent** (comparison group) **vs adherent.**

	B	SE	Wald	p	OR	95% CI OR
**Multivariate**						
Rurality	-0.91	0.64	2.05	0.152	0.40	[.12,1.40]
Intention<12	-1.69	0.84	4.09	0.043*	0.18	[.04,.95]
Intention=12	-1.70	0.74	5.31	0.021*	0.18	[.04,.78]
HADS Anxiety	-0.01	0.09	0.03	0.87	0.99	[.83,1.17]
HADS Depression	0.34	0.15	4.91	0.027*	1.40	[1.04,1.89]

* =significant at p<.05. ** Intention >12 used as comparison

Table S2: Multivariate logistic regression analyses of demographic, cognitive and emotional factors in people whose adherence **dropped-off** over time **(**comparison group) vs people who are adherent

	**B**	**SE**	**Wald**	**p**	**OR**	**95% CI OR**
**<u>Multivariate</u>**						
Action Plan	-0.14	0.13	1.13	0.289	0.87	[.68,1.12]
Self-efficacy	-0.05	0.05	1.47	0.225	0.95	[.87,1.03]
HADS depression	0.14	0.09	2.28	0.132	1.15	[.96,1.38]

* =significant at p<.05. ** Intention >12 used as comparison
