# Supplementary material for: Describing, predicting and explaining adherence to total skin self-examination (TSSE) in people with melanoma: a 12-month longitudinal study
Source: BMJ Open. 2022 Aug 30;12(8):e056755. doi: 10.1136/bmjopen-2021-056755 (PMC9438032; doi:10.1136/bmjopen-2021-056755)
Supplement: Author's manuscript [file bmjopen-2021-056755.draft_revisions.pdf]

BMJ Open is committed to open peer review. As part of this commitment we make the peer review history of every article we publish publicly available.

When an article is published we post the peer reviewers' comments and the authors' responses online. We also post the versions of the paper that were used during peer review. These are the versions that the peer review comments apply to.

The versions of the paper that follow are the versions that were submitted during the peer review process. They are not the versions of record or the final published versions. They should not be cited or distributed as the published version of this manuscript.

BMJ Open is an open access journal and the full, final, typeset and author-corrected version of record of the manuscript is available on our site with no access controls, subscription charges or pay-per-view fees (http://bmjopen.bmj.com).

If you have any questions on BMJ Open's open peer review process please email info.bmjopen@bmj.com

BMJ Open

Describing, predicting and explaining adherence to Total Skin Self-Examination (TSSE) in people with melanoma: a 12 month longitudinal study

Journal:	*BMJ Open*
Manuscript ID	bmjopen-2021-056755
Article Type:	Original research
Date Submitted by the Author:	27-Aug-2021
Complete List of Authors:	Allan, Julia; University of Aberdeen, Aberdeen Health Psychology Group<br>Johnston, D; University of Aberdeen<br>Johnston, Marie; University of Aberdeen, Aberdeen Health Psychology Group<br>Murchie, Peter; University of Aberdeen, Division of Applied Health Science
Keywords:	Dermatological tumours < ONCOLOGY, Adult oncology < ONCOLOGY, PREVENTIVE MEDICINE

SCHOLARONE™
Manuscripts

I, the Submitting Author has the right to grant and does grant on behalf of all authors of the Work (as defined in the below author licence), an exclusive licence and/or a non-exclusive licence for contributions from authors who are: i) UK Crown employees; ii) where BMJ has agreed a CC-BY licence shall apply, and/or iii) in accordance with the terms applicable for US Federal Government officers or employees acting as part of their official duties; on a worldwide, perpetual, irrevocable, royalty-free basis to BMJ Publishing Group Ltd ("BMJ") its licensees and where the relevant Journal is co-owned by BMJ to the co-owners of the Journal, to publish the Work in this journal and any other BMJ products and to exploit all rights, as set out in our licence.

The Submitting Author accepts and understands that any supply made under these terms is made by BMJ to the Submitting Author unless you are acting as an employee on behalf of your employer or a postgraduate student of an affiliated institution which is paying any applicable article publishing charge ("APC") for Open Access articles. Where the Submitting Author wishes to make the Work available on an Open Access basis (and intends to pay the relevant APC), the terms of reuse of such Open Access shall be governed by a Creative Commons licence – details of these licences and which Creative Commons licence will apply to this Work are set out in our licence referred to above.

Other than as permitted in any relevant BMJ Author's Self Archiving Policies, I confirm this Work has not been accepted for publication elsewhere, is not being considered for publication elsewhere and does not duplicate material already published. I confirm all authors consent to publication of this Work and authorise the granting of this licence.

For peer review only

Describing, predicting and explaining adherence to Total Skin Self-Examination (TSSE) in people with melanoma: a 12 month longitudinal study

Julia L. Allan[1*], j.allan@abdn.ac.uk

Derek W. Johnston[2], d.johnston@abdn.ac.uk

Marie Johnston[1], m.johnston@abdn.ac.uk

Peter Murchie[3], p.murchie@abdn.ac.uk

[1] Institute of Applied Health Sciences, University of Aberdeen, Aberdeen, UK

[2] School of Psychology, University of Aberdeen, Aberdeen, UK

[3] Centre for Academic Primary Care, University of Aberdeen, Aberdeen, UK

* Corresponding author. Institute of Applied Health Sciences, Health Sciences Building, University of Aberdeen, Foresterhill, Aberdeen. AB25 2ZD. +44(0)1224 438103

Keywords: melanoma; total skin self examination; adherence

Wordcount: 3,992

For peer review only

ABSTRACT

Objectives: To describe trajectories in melanoma survivors' adherence to monthly total skin self-examination (TSSE) over 12 months, and to investigate whether adherence trajectories can be predicted from demographic, cognitive or emotional factors at baseline.

Design: A longitudinal observational study nested within the intervention arm of the ASICA randomised controlled trial.

Setting: Follow up secondary care in Aberdeen and Cambridge UK.

Participants: n=104 adults (48M/56F; mean age 58.83 years, SD 13.47, range 28-85 years; mean SIMD score 8.03, SD 1.73, range 2-10) who had been treated for stage 0-IIC primary cutaneous melanoma in the preceding 60 months and were actively participating in the intervention arm of the ASICA trial.

Interventions: All participants were using the ASICA intervention – a tablet-based intervention designed to support monthly TSSE.

Primary and secondary outcome measures: The primary outcome was adherence to guideline recommended (monthly) TSSE over 12 months. This was determined from time-stamped TSSE data recorded by the ASICA intervention app.

Results: Latent growth mixed models identified 3 TSSE adherence trajectories (adherent - 41%; drop-offs -35%; and non-adherent -24%). The non-adherent were less likely to intend to perform TSSE as recommended, intending to do it more frequently (OR=0 .21, (95% CI 0.06, 0.81, p=.023) and were more depressed (OR =1.31 (CI , 1.06, 1.61, p=.011) than the adherent. The drop-offs had less well developed action plans (OR=0.78, CI 0.63,0.96, p=.016) and lower self-efficacy about TSSE (OR =0.92, CI 0.86,0.99, p=.028) than the adherent.

Conclusions: Adherence to monthly TSSE in individuals treated for melanoma can be reliably differentiated into three distinct trajectories over time: adherent, drop-off and non-adherent. Collecting information about intentions to engage in TSSE, depression level, self-efficacy and/or action planning at outset may help to identify those who would benefit from additional early, or later top-up intervention.

Trial registration: ClinicalTrials.gov Identifier: NCT03328247. Registered 01/11/2017.

For peer review only

ARTICLE SUMMARY

Strengths and limitations of this study

- A large sample of people treated for primary cutaneous melanoma were observed over 12 months to determine frequency and maintenance of adherence to recommended total skin self-examination (TSSE) over time
- TSSE adherence was objectively recorded via interactions with an app
- A 3-class latent growth mixed model produced adherence classifications that were highly satisfactory statistically and interpretable
- The sample showed limited variance in socioeconomic status, tending to be more affluent than the general population
- Quality of TSSE (i.e. thoroughness) was not a focus of this study and should be explored in future studies

For peer review only

INTRODUCTION

Melanoma is a relatively common skin cancer. There are more than 16,000 new cases of melanoma each year in the UK (1). Over the last 30 years, incidence rates have more than doubled (1), and rates are projected to continue increasing in future. Survival rates in melanoma are best predicted by tumour thickness at diagnosis (e.g. 2,3) so early detection is vital. If melanoma is diagnosed at stage I, 99% of patients are still alive after 5 years compared to 27% when melanoma is diagnosed at stage IV (4).

Post-diagnosis, around 20% of melanoma patients will experience a recurrence (5) and 4-8% will develop a new primary tumour (6), most commonly within 5 years. As the majority of melanomas are readily visible on the skin's surface and show observable changes over time, clinical guidelines for the management of potential recurrence emphasise the importance of regular skin examination (7,8). Gold standard total skin self-examination (TSSE) involves regular 'careful, deliberate and purposeful examination of the skin' (9), ideally monthly (10).

Most recurrences and new primaries are detected between scheduled follow ups by patients during TSSE (11-15). Individuals who detect a recurrence between scheduled follow ups have improved survival rates, likely because the melanomas are thinner / at an earlier stage on detection (16,17). Indeed, it has been estimated that regular TSSE may reduce mortality from melanoma by as much as 63% (9).

Despite the clear benefits of TSSE, adherence amongst people with melanoma is typically suboptimal. Surveys suggest that around 12% of patients never engage in recommended TSSE (18) having never been directly advised to perform it (19) or advised to perform it but not shown how (20). When TSSE is performed, it is often insufficiently frequent: a recent systematic review of 30 studies demonstrated that only a minority of melanoma patients (16-24%) adhere to recommendations by performing TSSE monthly (21).

Interventions developed to support engagement in more regular TSSE, have demonstrated initially positive results. For example, in one randomised controlled trial with over 1,300 melanoma patients, those randomised to an intervention that supported TSSE with instructions, prompts, support and feedback performed significantly more TSSEs at 2, 6 and 12 months than the control group (22). However, while more skin surgeries were performed in the intervention than control group at 6 months, these were not associated with additional malignant diagnoses and there was no difference in surgeries between groups by 12 months. Similarly, in a trial testing the effects of a video intervention designed to increase TSSE, the intervention increased prevalence of TSSE at 7 months, but by 13 months, prevalence of

For peer review only

TSSE was comparable in the intervention and control groups (23) suggesting that the beneficial effects dissipated over time.

As sustained adherence to monthly TSSE is 'crucial' in melanoma care (24), it has been suggested that regular reminders and booster educational sessions should be included in interventions to try and minimise drop off in optimal TSSE over time (24). The same authors report that self-efficacy (i.e. confidence in ability to perform a TSSE) and intentions to perform a TSSE are two of the strongest psychosocial predictors of engagement in TSSE (25) and that as such, should be included in TSSE interventions. Specifically, they suggest that TSSE interventions should incorporate clear demonstrations of how to perform TSSE and concrete instructions about what to do if a suspicious change is detected (to enhance self-efficacy) and should include techniques such as implementation intentions (short specific, goal focused plans, 26) to help people convert their intentions into actions.

One intervention which includes all of the content suggested to optimise TSSE over time is ASICA (Achieving Self-Directed Integrated Cancer Aftercare), a digital intervention designed to support high-quality, regular TSSE in people with cutaneous melanoma (27,28). The details of intervention development are reported elsewhere (27) but in brief, ASICA is a tablet (Android) delivered behavioural intervention based on the Information-Motivation-Behaviour model (29) and including behaviour change techniques based on Control Theory (30) and Implementation Intentions (26) to support engagement in TSSE. The ASICA intervention prompts and supports TSSE in people with melanoma by; 1) motivating people to perform TSSE; 2) building confidence (self-efficacy) in the ability to perform TSSE; 3) prompting monthly TSSE; 4) supporting thorough TSSE with body area guides; 5) prompting planning of TSSE and 6) prompting timely action if skin changes are detected.

ASICA was recently evaluated in a randomised controlled trial over 12 months with 240 melanoma patients (28,31) to determine the intervention's effect on TSSE behaviour and psychological wellbeing. One objective of the trial was to collect detailed data on the frequency and maintenance of TSSE behaviour over time in order to investigate whether clinically relevant patterns in adherence behaviour can be detected and predicted early on, and whether this knowledge can be used to optimise future interventions.

The present study aims to answer three research questions;

1. *What* does adherence to TSSE over 12 months look like in terms of frequency and maintenance?

For peer review only - http://bmjopen.bmj.com/site/about/guidelines.xhtml

2. *Who* (in sociodemographic terms) shows optimal and suboptimal TSSE adherence over 12 months?

3. *Why* does optimal and suboptimal TSSE adherence occur? That it is, can TSSE adherence be predicted from socio-cognitive (intentions, self-efficacy, action plans) and/or emotional (recurrence worries, anxiety, depression) factors?

For peer review only

METHODS

Design

A longitudinal study of objectively recorded TSSE adherence in people with melanoma over 12 months. The study was embedded within the intervention arm of the recently completed ASICA randomised controlled trial (27,28).

Ethics Approval

The project received full approval from the North of Scotland Research Ethics Committee on 28th April 2017 (17/NS/0040). Written informed consent was obtained from all study participants.

Patient and Public Involvement

A detailed pilot study was conducted during the development of the ASICA project to ascertain patients' priorities, experiences and preferences. Interviews were carried out with 19 potential recipients of the ASICA intervention, and these interviews informed the development of the study research questions and selection of outcome measures. Patients were not directly involved in the design of the study but did inform the design via participation in the pilot study interviews. The burden of the ASICA intervention was assessed by patients in a qualitative sub study. Two patient representatives sat on the trial steering group, feeding into plans for recruitment and dissemination. The results of the project will be disseminated to all participants (other than those who opted out) via a postal newsletter.

For peer review only - http://bmjopen.bmj.com/site/about/guidelines.xhtml

ASICA Trial and Intervention

The ASICA trial was a two-arm, multi centre randomised controlled trial designed to test the effects of the ASICA intervention on TSSE frequency and psychological wellbeing in adults treated for a stage 0-IIC (32) primary cutaneous melanoma. Participants were randomly allocated on a 1:1 basis to the ASICA intervention (intervention plus usual care) or control (usual care only) group. Intervention participants received the app-based ASICA intervention. The app included information about the importance of monthly TSSE, instructional videos demonstrating TSSE, a digital map of the patient's own skin, a checkbox list of body parts to check, prompts to plan TSSE and the capability to send photographs of suspicious skin lesions to a dermatology nurse practitioner for review. As part of the intervention, participants were prompted monthly (i.e. in line with guideline frequency) to conduct a TSSE.

Participants

Participants were n=104 adults (48M/56F; mean age 58.83 years, SD 13.47, range 28-85 years; mean SIMD score 8.03, SD 1.73, range 2-10) who had been treated for stage 0-IIC primary cutaneous melanoma in the preceding 60 months and who were actively engaged in the intervention arm of the ASICA trial between 2018 and 2020.

Measures

TSSE adherence. TSSE adherence was determined from data collected by the ASICA intervention app. After completing a practice, participants were prompted every 30 days for the next 12 months to complete and report a TSSE. Adherence to TSSE guidelines was determined by assessing via the time-stamped data collected by the ASICA app whether participants had completed at least one TSSE during each 30 day period following the receipt of a prompt (i.e. had engaged in TSSE at least monthly). Each period was coded 1 (at least one report received) or 0 (no report received), with possible scores ranging from 0 (totally non-adherent) to 13 (totally adherent, 1 practice + 12 monthly TSSEs).

Demographic, cognitive and emotional factors were measured in a baseline questionnaire administered at the start of the ASICA trial.

Demographic factors.

For peer review only - http://bmjopen.bmj.com/site/about/guidelines.xhtml

Age in years and gender were self-reported. Socioeconomic status was coded 1 (most deprived) to 10 (least deprived) by linking postcodes to indices of deprivation (Scottish Index of Multiple Deprivation; English indices of deprivation (33,34). Rurality/Urbanity was coded from 1 (most urban) to 10 (most rural) (35,36).

Cognitive factors.

Intention towards engaging in TSSE was assessed with the item "In the next 12 months, do you intend to check your skin for early signs of skin cancer? [yes/no]…. If yes, how many times? [insert number]". In order to map intentions onto guideline behaviour, this item was scored categorically as <12 (intention below guideline), =12 (guideline intention) or >12 (intention above guideline).

Self-efficacy towards TSSE was assessed with 4 items rated on a 10-point scale from 'not at all confident' to 'highly confident': "How confident are you that…….you can check your own skin correctly?; that you will find time in the next 12 months to check your own skin?; that you will remember to check your own skin at least once a month?; that if you find a spot or mole of concern that you will take appropriate action?"

Action Planning about TSSE was assessed with 2 items rated on a 5-point scale from 'strongly agree to 'strongly disagree': "I have made plans about when to examine my own skin" and "I have made plans about where I will be when I examine my own skin".

Emotional factors.

Anxiety and depression were measured using the 14 item Hospital Anxiety & Depression Scale (HADS; 37), a valid measure with adequate internal consistency (38). Anxiety and depression were scored separately (from 0-4), with higher scores indicating greater distress.

Melanoma specific worries were assessed with the 4 item Melanoma Worry Scale (39). Items were rated on a 5 point scale from 'not at all' to 'almost all the time' and assessed the frequency and impact of worries about possible recurrence.

Analysis

Latent group mixture modelling (LGMM) was carried out with Mplus V8.4 (40) to establish if there were systematically different classes of TSSE over the 12 month measurement period.

For peer review only - http://bmjopen.bmj.com/site/about/guidelines.xhtml

A series of models were estimated from the TSSE adherence data with 1 to 5 latent classes, and were compared on the Bayesian information criterion, entropy values and number of participants per class. Class membership was obtained solely on the basis of the adherence data (i.e., covariates were not included in the modelling exercise) and the resulting classification information was then combined with the other information to explore the differences between the classes using the classical three-step method (41). In the present analysis, the LGMM identified 3 classes of adherence behaviour (adherent, drop-offs and non-adherent). Logistic regressions were then conducted using SPSS v25 to predict membership of these 3 classes from demographic (age, gender, socioeconomic status, rurality), cognitive (intentions, self-efficacy and action plans about TSSE) and emotional (anxiety, depression and melanoma worry) factors. In these logistic regression analyses, the dependent variable was dummy coded (with the adherent coded as 0 and the non-adherent or drop-off coded as 1). Categorical intention scores were treated as unordered categories. Missing data, which were <4% on all measures other than intention and 11.5% for intention, were dealt with by item deletion. Intention analyses were re-run with missing values imputed and results did not change.

RESULTS

What does adherence to TSSE over 12 months look like in terms of frequency and maintenance?

Of the 104 participants, 12 did not submit any TSSE reports using ASICA and the remainder submitted between 1 and 13 (median 6.5, IQR 9.75).

LGMM was tested with 1 to 5 latent groups. The lowest (best) Bayesian Information Criterion (BIC) was for a 3 class solution (1318). The entropy score (a measure of the quality of the classification) for the 3 class solution was also highly satisfactory (.906). The number of participants per class was substantial (25/36/34) and the classes interpretable. The three class solution was therefore selected for further study.

Figure 1 shows the LGMM estimated and observed values plotted over time and illustrates the 3 distinct classes identified. The largest class are the 'adherents' (n=43) who achieve over 90% adherence across the study period and show no significant decrease over time (p=.188). The next class, the 'drop-offs' (n=36) are those who start well, particularly after the first

For peer review only - http://bmjopen.bmj.com/site/about/guidelines.xhtml

prompt but whose TSSE adherence declines steadily over 12 months (p< .001) until only around 20% are providing the required reports by the final month. The final group, the 'non-adherents' (n=25) start poorly, submitting only 28% of reports in the first month, and decrease rapidly such that they are providing virtually no reports by 6 months (p=.022). An overview of the characteristics of participants in the three TSSE adherence classes can be found in Table 1. The accuracy of the classification (likely versus actual classification) was high (adherent =0.98; drop-offs =0.93; non-adherents = 0.96). Figure 2 shows the cumulative number of months in which a TSSE report was submitted for individual participants grouped by latent class.

Table 1: Demographic, cognitive and emotional factors in the three adherence groups

	Adherent	Drop-Off	Non-Adherent
N	43	36	25
Age in years (M, SD)	60.45 (11.77)	57.12 (13.31)	58.50 (16.38)
Gender (M/F)	16/27	20/16	12/13
Rurality (urban/rural)	21/22	23/13	18/7
Social Deprivation (M, SD)	7.98 (1.50)	8.17 (1.72)	7.92 (2.14)
Action Planning (M, SD)	6.86 (2.10)	5.56 (2.47)	6.25 (2.42)
Self-Efficacy (M, SD)	32.32 (5.89)	28.85 (7.04)	30.50 (6.90)
Intentions (n)*			
<12 times	13	15	6
=12 times	22	11	7
>12 times	6	3	9
HADS Anxiety (Mean, SD)	4.23 (3.99)	5.33 (3.84)	6.13 (4.95)
HADS Depression (Mean, SD)	1.93 (2.19)	3.14 (3.45)	3.75 (3.05)
Melanoma Worry (Mean, SD)	8.33 (3.52)	8.73 (3.59)	8.75 (3.90)

* Only 92/104 participants completed the intention measure

Who (in sociodemographic terms) engages in optimal and suboptimal TSSE adherence and Why does optimal and suboptimal TSSE adherence occur?

For peer review only - http://bmjopen.bmj.com/site/about/guidelines.xhtml
For peer review only

The results of the logistic regression analyses predicting adherence class (adherent; drop-off; non-adherent) from the demographic, cognitive and emotional measures are shown in Table 2 (comparison of non-adherent and adherent) and Table 3 (comparison of drop-offs and adherent).

Table 2: Logistic regression analyses of demographic, cognitive and emotional factors in **non-adherent** (comparison group) **vs adherent.**

	B	SE	Wald	p	OR	95% CI OR
**Univariate**						
Age	0.01	0.02	0.33	0.566	0.99	[.95,1.03]
Gender	0.44	0.51	0.76	0.385	1.56	[.57,4.23]
SIMD	-0.02	0.14	0.02	0.9	0.98	[.74,1.30]
Rurality	-0.99	0.54	3.37	0.066	0.37	[.13,1.07]
Intention <12 **	-1.18	0.72	2.67	0.103	0.31	[.08,1.27]
Intention =12**	-1.55	0.68	5.17	0.023*	0.21	[.06,.81]
Action plan	-0.13	0.12	1.17	0.28	0.88	[.70,1.11]
Self-efficacy	-0.04	0.05	1.18	0.28	0.96	[.88,1.04]
HADS anxiety	0.1	0.06	2.76	0.096	1.10	[.98,1.24]
HADS depression	0.27	0.11	6.43	0.011*	1.31	[1.06,1.61]
MWS	0.04	0.07	0.23	0.633	1.04	[0.90,1.19]

* =significant at p<.05. ** Intention >12 used as comparison

Table 3: Logistic regression analyses of demographic, cognitive and emotional factors in **drop-offs (**comparison group) **vs adherent**

	B	SE	Wald	p	OR	95% CI OR
**Univariate**						
Age	-0.02	0.02	1.38	0.241	0.98	[.94,1.02]
Gender	0.75	0.46	2.63	0.105	2.11	[.86,5.2]
SIMD	-0.08	0.14	0.28	0.597	1.08	[.81,1.43]
Rurality	-0.62	(0.46)	1.78	0.182	0.54	[.22,1.33]
Intention <12**	0.84	0.8	1.09	0.297	2.31	[.48,11.12]
Intention =12**	0	0.8	0	1	1	[.21,4.78]
Action plan	-0.25	0.11	5.77	0.016*	0.78	[.63,.96]
Self-efficacy	-0.08	0.04	4.82	0.028*	0.92	[.86,.99]

For peer review only

HADS Anxiety	0.07	0.06	1.52	0.217	1.08	[.96,1.21]
HADS Depression	0.16	0.09	3.19	0.074	1.17	[.98,1.40]
MWS	0.04	0.07	0.26	0.61	1.06	[.91,1.18]

* =significant at p<.05. ** Intention >12 used as comparison

Age, sex and SIMD did not significantly predict adherence class in any analysis, indicating that demographic factors alone are not sufficient to predict different adherence trajectories over time. However there was a tendency for rural participants to be more adherent.

In univariate comparisons of *cognitive factors*, non-adherents were less likely to intend to carry out TSSE monthly than adherents, instead reporting (potentially unrealistic) intentions to perform TSSE more often than monthly. Non–adherents did not differ from adherents in terms of action plans or self-efficacy towards TSSE. Drop-offs did not differ significantly from adherents in their TSSE intentions. However the drop-offs reported lower levels of action planning and self-efficacy than the adherent. When *emotional factors* were compared, the non-adherents had significantly higher depression scores than the adherents with the drop offs intermediate but not reliably different from the adherents. Anxiety was slightly higher in the non-adherent group but this was not significant at p<.05. Melanoma worries did not significantly differ between any of the adherence groups.

DISCUSSION

Distinct patterns in TSSE adherence emerged in participants using the ASICA intervention over 12 months. While 41% of participants ('adherents') consistently completed >90% of guideline recommended TSSEs over 12 months, the remaining 59% showed one of two patterns of suboptimal adherence. Approximately 35% ('drop-offs') initially adhered well but showed steady and significant declines in TSSE adherence over time until only around a fifth were conducting TSSEs by 12 months. The remaining 24% ('non-adherents') consistently failed to engage in monthly TSSEs, with only a minority (around a third) conducting a TSSE in the first month and virtually none completing one from 6 months onwards. One of the

For peer review only

aims of the present study was to determine whether participants who would go on to display suboptimal adherence could be identified at baseline so that they could be better supported in future at intervention outset. To this end, adherence trajectories were predicted from a range of easily measurable and theoretically relevant demographic, cognitive and emotional factors. None of the demographic factors assessed in the current study (age, gender, socioeconomic status, rurality) differentiated between classes of TSSE adherence behaviour, although there was a slight tendency for those living in urban areas to be more non-adherent. Of the cognitive and emotional factors studied, intentions about TSSE and depression reliably distinguished between the adherent and non-adherent, and self-efficacy and action planning distinguished the drop-offs from the adherent.

For peer review only

Overall Adherence

In terms of overall adherence, the present results suggest that the ASICA intervention may have had a beneficial effect on TSSE adherence as 41% of participants in the present study showed optimal adherence over a year compared to 16-24% on average across the 30 TSSE studies included in a recent systematic review (21). However, the results also demonstrate that even with the theory and evidence-based support provided by ASICA, TSSE adherence is still suboptimal in the majority of participants.

Predictors of Adherence

Demographic factors were not sufficient to predict adherence trajectory. However, it should be noted that participants in this study showed extremely limited variance in socioeconomic status, tending to be more affluent than the general population. This restricted range likely reflects the combined effects of melanoma incidence being higher in more affluent individuals (e.g. 42), and trial participation being skewed towards individuals from higher socioeconomic groups, and limits the conclusions that can be drawn about socioeconomic status.

When cognitive factors (intentions about TSSE, self-efficacy towards TSSE and plans about when and where to perform TSSE) were examined, the data suggested that different cognitive processes may be involved in the two suboptimal patterns of adherence observed ('non-adherent' and 'drop-offs'). 'Non-adherents' were significantly more likely to have high and potentially unrealistic intentions about TSSE than the adherent: with many intending to perform TSSEs more frequently than monthly. Neither TSSE self-efficacy nor action

For peer review only - http://bmjopen.bmj.com/site/about/guidelines.xhtml

planning significantly differentiated the non-adherent from the adherent. In contrast, the opposite pattern was found to differentiate the 'drop-offs' from the adherent: the two groups did not significantly differ in their intentions about TSSE but the drop-offs showed significantly lower levels of action planning and self-efficacy than the adherent. The observed pattern of results fits well with theoretical models of health behaviour change such as the Health Action Process Approach (HAPA; 43). HAPA explicitly distinguishes between motivational processes (such as intentions) involved in the early goal-setting phase of behaviour change, and volitional processes (such as self-efficacy and planning) involved in the later goal-pursuit phase of behaviour change. In the present study, the participants who were least likely to 'get started' (i.e. the non-adherents) showed measurable differences in motivational processes (intentions) relative to the adherent. Similarly, those who started well (had motivation) but then had issues with maintaining the volitional aspects of regular TSSE over time (i.e. the drop-offs) showed lower levels of self-efficacy and planning relative to the adherent. Cognitive factors were on average less predictive of TSSE behaviour in the present study than in other melanoma studies (25), possibly because the ASICA intervention was designed to boost intentions, self-efficacy and planning. This may have reduced natural variance in these constructs, limiting their predictive power.

The analysis of the emotional factors (anxiety, depression and melanoma specific worries) indicated that while the adherent consistently had the 'best' scores (i.e. lowest anxiety, lowest depression, fewest worries about recurrence), the only factor that significantly differentiated the adherent from the non-adherent was depression. Drop-offs in contrast, did not significantly differ from the adherent on any of the emotional factors. It is noteworthy that melanoma specific worries were remarkably consistent across adherence groups and did not predict TSSE behaviour. Fears about recurrence are often suggested as a likely predictor of TSSE behaviour (i.e. that people worried about recurrence will check their skin more frequently, potentially more frequently than recommended), and recent evidence suggests that people who are more worried about recurrence are more likely to conduct TSSE and to conduct more thorough TSSE (44). The present results were not in line with this, suggesting that recurrence fears were unrelated to TSSE adherence over 12 months.

Clinical Implications

The present results suggest that routinely screening for fear of recurrence will not helpfully discriminate melanoma patients in terms of their likely future TSSE adherence. Making baseline assessments of participants' intentions about TSSE and level of depression however

For peer review only

may serve to highlight the group most likely to be non-adherent. Intentions, if suboptimal, can in theory be strengthened with three behaviour change techniques: giving information about the health consequences of TSSE, setting goals about completing TSSE and providing an incentive for reaching the outcome (45). Similarly, depression might be reduced by helping patients to more effectively manage negative emotions, e.g. through stress management techniques (45). It is important to note however that the effect sizes in the present study were small so the potential clinical benefit of this approach would be limited.

Identifying those who start well but falter (the 'drop-offs') is challenging. While measures of self-efficacy and action planning differentiated drop-offs from the adherent on average, they would not reliably identify people on this adherence trajectory at baseline (e.g. baseline self-efficacy scores in the present data set would correctly identify only 46% of drop-offs). Instead, adherence behaviour would have to be monitored for 6 months (in the present data), to identify 75% of the drop-offs (as those who have completed <4 of the recommended 6 TSSEs). Top up interventions could be targeted at this point: action planning might be addressed by prompting people to make 'if-then' plans (26) and self-efficacy might be enhanced by methods such as verbal persuasion and focusing on past success (e.g. in adhering in the early stages) (45). If top-up interventions were to be used, it would be possible in the present data to accurately identify 92% of the non-adherent and 100% of the adherent at 3 months on the basis of TSSEs completed (0-1 vs 2-3).

Strengths and Limitations

The present study has several methodological strengths. It uses objectively recorded TSSE behaviour rather than self-reports, and we can therefore be confident that TSSE took place at the times indicated. The study also followed participants over a full 12 months and tested both emotional and socio-cognitive models of behaviour. However, as discussed above, one notable limitation of the current study is that participants came almost exclusively from the less deprived end of the socioeconomic spectrum. While broadly representative of the clinical population (melanoma being more common in high SES individuals) the results reported are not representative of the general population. This raises an important point about whether digital interventions like ASICA would exacerbate existing health inequalities if uptake (and therefore potential to benefit) is higher in individuals from less deprived sectors of the population (cf.46,47), or whether they would usefully 'free up' face to face resources for those least likely to engage with remote healthcare

For peer review only

Future Directions

Future studies should investigate in more detail methods for early detection of those least likely to be adherent. Based on our data, interventions which boost action planning and self-efficacy at 3 and 6 months might be beneficial in reducing the number of drop-offs while an intervention prior to starting monthly TSSE to address motivation and depression might reduce the number of people who go onto be non-adherent. Detection of sub-optimal adherence on an individual level would require real time measurement and more dynamic and individualised assessment in order to model the thresholds beyond which optimal adherence can be reliably distinguished from adherence which is likely to decline in future. Future studies should also extend the present work on the frequency and maintenance of TSSE to TSSE thoroughness. Studies which have investigated the thoroughness of TSSE, suggest that only 7.5-14% of patients check their whole body while performing TSSE (44,48) and only 9-13% use a mirror or ask another person to help them check areas that are difficult to see (48). Melanoma can recur anywhere on the body and more thorough SSE is associated with detection of earlier stage, more survivable lesions (49).

In conclusion, the present study demonstrates that adherence to recommended monthly TSSE in individuals treated for melanoma can be reliably differentiated into three distinct trajectories over time: adherent, drop-off and non-adherent. Collecting information about intentions to engage in TSSE, depression level, self-efficacy and/or action planning at outset may help to distinguish between those who go on to engage in optimal, declining or consistently suboptimal adherence.

Funding statement

This work was supported by a grant from a Cancer Research UK Population Research Committee project award (C10673/A21685). The views and opinions expressed herein are those of the authors and do not necessarily reflect those of Cancer Research UK. The funder (through their peer review and funding board review process) approved the study proposal but had no role in the collection, analysis, or interpretation of data, or writing of the report.

Competing interests statement

None of the authors have competing interests to declare.

Author contributions

For peer review only - http://bmjopen.bmj.com/site/about/guidelines.xhtml

All authors conceived of and designed the study. DWJ analysed the data. JA drafted the manuscript. All authors contributed to interpretation of the results and revised the manuscript.

Data sharing statement

Data are available on request.

Figure Legends

Figure 1: The 3 classes of adherence to TSSE over time showing LGMM estimated and observed values

Figure 2: Cumulative number of TSSEs conducted (y axis) over 13 successive 30 day periods (x axis) in individual participants classed as 'adherent', 'drop-off', and 'non-adherent'

For peer review only

References

1. Cancer Research UK. Melanoma skin cancer statistics 2015-17. Retrieved from https://www.cancerresearchuk.org/health-professional/cancer-statistics/statistics-by-cancer-type/melanoma-skin-cancer

2. Baade PD, English DR, Youl PH, *et al*. The relationship between melanoma thickness and time to diagnosis in a large population-based study. *Arch Dermatol* 2006;142:1422–7. https://doi.org/10.1001/archderm.142.11.1422.

3. Balch CM, Gershenwald JE, Soong SJ, *et al*. Final version of 2009 AJCC melanoma staging and classification. *J Clin Oncol* 2009;27:6199–206. https://doi.org/10.1200/jco.2009.23.4799.

4. American Cancer Society. 2021. Retrieved from https://www.cancer.org/cancer/melanoma-skin-cancer/detection-diagnosis-staging/survival-rates-for-melanoma-skin-cancer-by-stage.html

5. Francken AB, Accortt NA, Shaw HM, *et al*. Follow-up schedules after treatment for malignant melanoma. *Br J Surg* 2008;95:1401-1407. https://doi.org/10.1002/bjs.6347

6. Di Fronzo LA, Wanek LA, Elashoff R *et al*. Increased incidence of second primary melanoma in patients with a previous cutaneous melanoma. *Ann Surg Oncol* 1999;6:705711.

7. National Institute for Health and Care Excellence (NICE). Melanoma: Assessment and management (NG14; 2015). Retrieved from https://www.nice.org.uk/guidance/ng14

8. Health Improvement Scotland. SIGN QRG 146 Cutaneous Melanoma. 2016. Retrieved from https://www.sign.ac.uk/assets/qrg146.pdf

9. Berwick M, Begg CB, Fine JA. Screening for cutaneous melanoma by skin self-examination. *J Natl Cancer Inst* 1996;88:17–23.

10. Watts CG, Dieng M, Morton RL, *et al*. Clinical practice guidelines for identification, screening and follow-up of individuals at high risk of primary cutaneous melanoma: a systematic review. *Br J Dermatol* 2015;172:33–47. https://doi.org/10.1111/bjd.13403.

11. Dicker TJ, Kavanagh GM, Herd RM, *et al*. A rational approach to melanoma follow-up in patients with primary cutaneous melanoma. Scottish Melanoma Group. *Br J Dermatol* 1999;140:249–254. https://doi.org/10.1046/j.1365-2133.1999.02657.x

12. Meyers MO, Yeh JJ, Frank J, *et al* (2009). Method of detection of initial recurrence of stage II/III cutaneous melanoma: analysis of the utility of follow-up staging. *Ann Surg Oncol*, 2009;16:941–947. https://doi.org/10.1245/s10434-008-0238-y

13. Auckland R, Wassell P, Hall S, *et al.* Exploring patterns of recurrent melanoma in Northeast Scotland to inform the introduction a digital self-examination intervention. *BMC Dermatol* 2014;14:4. https://doi.org/10.1186/1471-5945-14-4

14. Brady MS, Oliveria SA, Christos PJ, *et al*. Patterns of detection in patients with cutaneous melanoma. *Cancer* 2000;89:342–347.

15. Swetter SM, Pollitt RA, Johnson TM, *et al*. Behavioral determinants of successful early melanoma detection: role of self and physician skin examination. *Cancer* 2012;118:3725–34. https://doi.org/10.1002/cncr.26707.

16. Moore Dalal K, Zhou Q, Panageas KS*, et al*. Methods of detection of first recurrence in patients with stage I/II primary cutaneous melanoma after sentinel lymph node biopsy. *Ann Surg Oncol* 2008;15:2206-2214. doi: 10.1245/s10434-008-9985-z

17. Paddock LE, Lu SE, Bandera EV, *et al*. Skin self-examination and long-term melanoma survival. *Melanoma Res* 2016;26:401–408.

18. Hull PR, Piemontesi NG, Lichtenwald J. Compliance with self-examination surveillance in patients with melanoma and atypical moles: an anonymous questionnaire study. *J Cutan Med and Surg* 2011;15:97–102. https://doi.org/10.2310/7750.2011.10011

19. Körner A, Coroiu A, Martins C, *et al*. Predictors of skin self-examination before and after a melanoma diagnosis: the role of medical advice and patient's level of education. *Int Arch Med* 2013;6:8. https://doi.org/10.1186/1755-7682-6-8

20. Manne S, Lessin S. Prevalence and Correlates of Sun Protection and Skin Self-Examination Practices Among Cutaneous Malignant Melanoma Survivors. *J Behav Med* 2006;29**:**419–434. https://doi.org/10.1007/s10865-006-9064-5

21. Bhurosy T, Heckman CJ, Riley M. Prevalence and correlates of skin self-examination behaviors among melanoma survivors: a systematic review, *Transl Behav Med* 2020;10:1120–1133, https://doi.org/10.1093/tbm/ibaa003

For peer review only - http://bmjopen.bmj.com/site/about/guidelines.xhtml

22. Weinstock MA, Risica PM, Martin RA, *et al*. Melanoma early detection with thorough skin self-examination: the "Check It Out" randomized trial. *Am J Prev Med* 2007; 32:517–524. https://doi.org/10.1016/j.amepre.2007.02.024

23. Janda M, Neale RE, Youl P, *et al*. Impact of a video-based intervention to improve the prevalence of skin self-examination in men 50 years or older: the randomized skin awareness trial. *Arch Dermatol* 2011;147:799–806. https://doi.org/10.1001

24. Coroiu A, Moran C, Bergeron C, *et al.* Short and long-term barriers and facilitators of skin self-examination among individuals diagnosed with melanoma. *BMC Cancer* 20**;**123. https://doi.org/10.1186/s12885-019-6476-5

25. Coroiu A, Moran C, Bergeron C, *et al* (2020a). Operationalization of skin self-examination in randomized controlled trials with individuals at increased risk for melanoma: A systematic review. *Pat Ed Couns*, 2020;*103*:1013–1026. https://doi.org/10.1016/j.pec.2019.12.009

26. Gollwitzer PM. Implementation intentions: strong effects of simple plans. *Am Psychol* 1999;54:493.

27. Murchie P, Allan JL, Brant W, *et al*. Total skin self-examination at home for people treated for cutaneous melanoma: development and pilot of a digital intervention. *BMJ Open* 2015;5:e007993

28. Murchie P, Masthoff J, Walter FM, *et al.* Achieving Self-Directed Integrated Cancer Aftercare (ASICA) in melanoma: protocol for a randomised patient-focused pilot trial of delivering the ASICA intervention as a means to earlier detection of recurrent and second primary melanoma. *Trials* 2019;20**:**318. https://doi.org/10.1186/s13063-019-3453-x

29. Fisher JD, Fisher WA. Changing AIDS-risk behavior. *Psychol Bull* 1992;111:455–74

30. Carver CS, Scheier MF. Attention and self-regulation: a control theory approach to human behaviour. New York: Springer; 1981.

31. Murchie P, Constable L, Hall S, *et al.* A randomised patient-focused trial of the Achieving Self-Directed Integrated Cancer Aftercare (ASICA) intervention for detection of recurrent and second primary melanoma: effect on psychological well-being and quality of life. In preparation.

32. Keung EZ, Gershenwald JE. The eighth edition American Joint Committee on Cancer (AJCC) melanoma staging system: implications for melanoma treatment and care. *Expert Rev Anticancer Ther*, 2018;18:775–784. https://doi.org/10.1080/14737140.2018.1489246

33. Scottish Government. Scottish Index of Multiple Deprivation. 2020. Retrieved from https://www.gov.scot/collections/scottish-index-of-multiple-deprivation-2020/

34. UK Government. National Statistics English indices of deprivation 2019. Retrieved from https://www.gov.uk/government/statistics/english-indices-of-deprivation-2019

For peer review only - http://bmjopen.bmj.com/site/about/guidelines.xhtml

35. Scottish Government. Urban Rural Classification 2016. Retrieved from https://www.gov.scot/publications/scottish-government-urban-rural-classification-2016/pages/2/

36. Office for National Statistics. Rural/urban classification 2011. Retrieved from https://www.ons.gov.uk/methodology/geography/geographicalproducts/ruralurbanclassifications

37. Zigmond AS, Snaith RP. The hospital anxiety and depression scale. *Acta Psychiat Scand* 1983;67:361-70.

38. Bjelland I, Dahl AA, Haug TT, et al. The validity of the Hospital Anxiety and Depression Scale: An updated literature review. *J Psychosom Res* 2002;52:69-77.

39. Moye MS, King SMC, Rice ZP, *et al*. Effects of total-body digital photography on cancer worry in patients with atypical mole syndrome. *JAMA Dermatol*, 2015;151:137–43. https://doi.org/10.1001/jamadermatol.2014.2229

40. Muthen LK, Muthen BO. Mplus User Guide. Eighth Edition. 1988-2017. Los Angeles, CA: Muthen & Muthen.

41. Wang J, Wang X. Structural Equation Modeling: Applications Using Mplus. Second Edition. 2020. John Wiley & Sons.

42. Johnson-Obaseki SE, Labajian V, Corsten MJ, *et al*. Incidence of cutaneous malignant melanoma by socioeconomic status in Canada: 1992-2006. *J Otolaryng Head Neck Surg* 2015;*44*:53. https://doi.org/10.1186/s40463-015-0107-1

43. Schwarzer R. Self-efficacy in the adoption and maintenance of health behaviours: Theoretical approaches and a new model. In: Schwarzer R, editor. Self-Efficacy: Thought Control of Action. Washington: Taylor and Francis; 1992. pp. 217–243.

44. Manne SL, Heckman CJ, Kashy D, *et al*. Prevalence and correlates of skin self-examination practices among cutaneous malignant melanoma survivors. *Prev Med Rep* 2020;19. https://doi.org/10.1016/j.pmedr.2020.101110.

45. Johnston M, Carey RN, Connell LE, *et al*. Development of an online tool for linking behavior change techniques and mechanisms of action based on triangulation of findings from literature synthesis and expert consensus. *Translat Behav Med* 2021;5:1049-1065. https://doi.org/10.1093/tbm/ibaa050

46. McAuley A. Digital health interventions: widening access or widening inequalities? *Pub Health* 2014;12:1118-1120.

47. Honeyman M, Maguire D, Evans H, *et al*. Digital technology and health inequalities: a scoping review. 2020. Cardiff: Public Health Wales NHS Trust.

48. Coups EJ, Manne SL, Stapleton JL, *et al*. Skin self-examination behaviors among individuals diagnosed with melanoma. *Melanoma Res* 2016;26:71–6. https://doi.org/10.1097/CMR.0000000000000204.

For peer review only - http://bmjopen.bmj.com/site/about/guidelines.xhtml

49. Pollitt RA, Geller AC, Brooks DR, *et al*. Efficacy of skin self-examination practices for early melanoma detection. *Cancer Epidemiol Biomark Prev* 2009;18:3018–23. https://doi.org/10.1158/1055-9965.epi-09-0310.

For peer review only

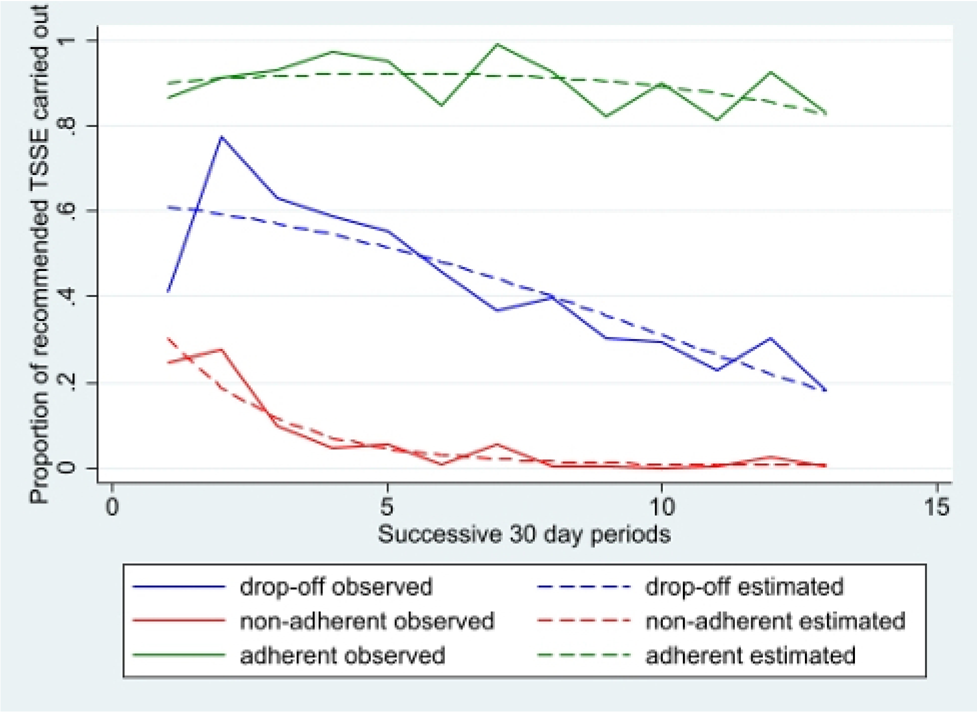

Figure 1: The 3 classes of adherence to TSSE over time showing LGMM estimated and observed values

172x125mm (72 x 72 DPI)

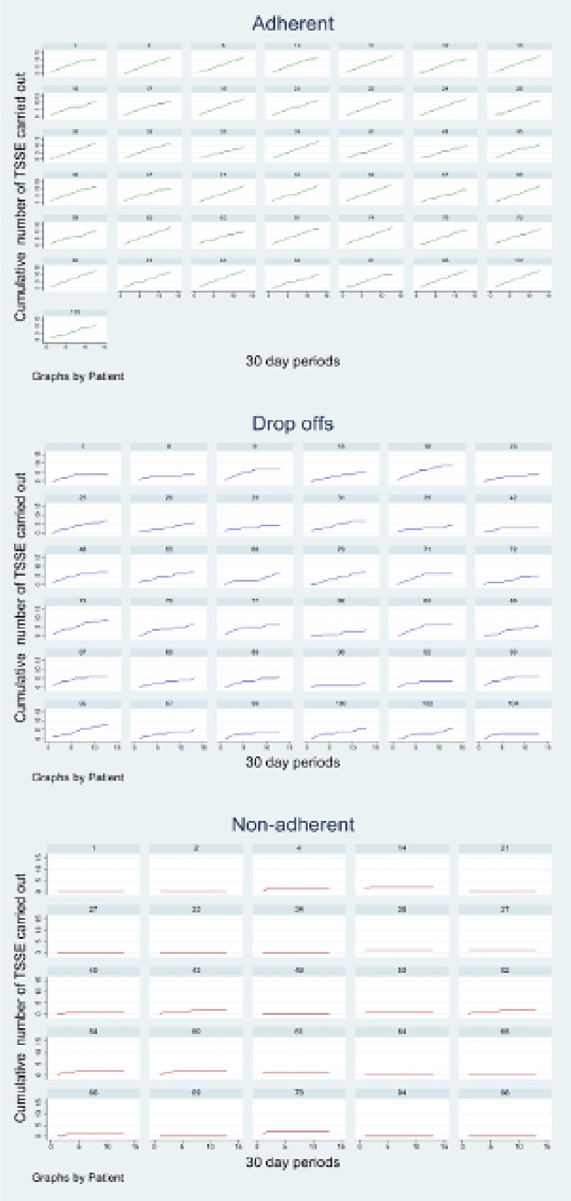

Figure 2: Cumulative number of TSSEs conducted (y axis) over 13 successive 30 day periods (x axis) in individual participants classed as 'adherent', 'drop-off', and 'non-adherent'

106x222mm (72 x 72 DPI)

STROBE 2007 (v4) checklist of items to be included in reports of observational studies in epidemiology*

Checklist for cohort, case-control, and cross-sectional studies (combined)

Section/Topic	Item #	Recommendation	Reported on page #
**Title and abstract**	1	(*a*) Indicate the study's design with a commonly used term in the title or the abstract	p2
		(*b*) Provide in the abstract an informative and balanced summary of what was done and what was found	p2
**Introduction**			
Background/rationale	2	Explain the scientific background and rationale for the investigation being reported	p4-5
Objectives	3	State specific objectives, including any pre-specified hypotheses	p5-6
**Methods**			
Study design	4	Present key elements of study design early in the paper	p5-6
Setting	5	Describe the setting, locations, and relevant dates, including periods of recruitment, exposure, follow-up, and data collection	p6-7
Participants	6	(*a*) *Cohort study*—Give the eligibility criteria, and the sources and methods of selection of participants. Describe methods of follow-up<br>*Case-control study*—Give the eligibility criteria, and the sources and methods of case ascertainment and control selection. Give the rationale for the choice of cases and controls<br>*Cross-sectional study*—Give the eligibility criteria, and the sources and methods of selection of participants	p7
		(*b*) *Cohort study*—For matched studies, give matching criteria and number of exposed and unexposed<br>*Case-control study*—For matched studies, give matching criteria and the number of controls per case	N/A
Variables	7	Clearly define all outcomes, exposures, predictors, potential confounders, and effect modifiers. Give diagnostic criteria, if applicable	p7-8
Data sources/ measurement	8*	For each variable of interest, give sources of data and details of methods of assessment (measurement). Describe comparability of assessment methods if there is more than one group	p7-8
Bias	9	Describe any efforts to address potential sources of bias	p7
Study size	10	Explain how the study size was arrived at	Stated in cited protocol (main RCT)
Quantitative variables	11	Explain how quantitative variables were handled in the analyses. If applicable, describe which groupings were chosen and why	p8-9
Statistical methods	12	(*a*) Describe all statistical methods, including those used to control for confounding	p8-9
		(*b*) Describe any methods used to examine subgroups and interactions	p8-9
		(*c*) Explain how missing data were addressed	p9
		(*d*) *Cohort study*—If applicable, explain how loss to follow-up was addressed	N/A

For peer review only

		Case-control study—If applicable, explain how matching of cases and controls was addressed<br>*Cross-sectional study*—If applicable, describe analytical methods taking account of sampling strategy	
		(*e*) Describe any sensitivity analyses	p9
**Results**			
Participants	13*	(a) Report numbers of individuals at each stage of study—eg numbers potentially eligible, examined for eligibility, confirmed eligible, included in the study, completing follow-up, and analysed	Table 1
		(b) Give reasons for non-participation at each stage	N/A
		(c) Consider use of a flow diagram	N/A
Descriptive data	14*	(a) Give characteristics of study participants (eg demographic, clinical, social) and information on exposures and potential confounders	Table 1
		(b) Indicate number of participants with missing data for each variable of interest	p9, Table 1
		(c) *Cohort study*—Summarise follow-up time (eg, average and total amount)	p7
Outcome data	15*	*Cohort study*—Report numbers of outcome events or summary measures over time	p9
		Case-control study—Report numbers in each exposure category, or summary measures of exposure	
		Cross-sectional study—Report numbers of outcome events or summary measures	
Main results	16	(*a*) Give unadjusted estimates and, if applicable, confounder-adjusted estimates and their precision (eg, 95% confidence interval). Make clear which confounders were adjusted for and why they were included	Tables 2 and 3
		(*b*) Report category boundaries when continuous variables were categorized	p8-9
		(*c*) If relevant, consider translating estimates of relative risk into absolute risk for a meaningful time period	N/A
Other analyses	17	Report other analyses done—eg analyses of subgroups and interactions, and sensitivity analyses	p7
**Discussion**			
Key results	18	Summarise key results with reference to study objectives	p10-12
Limitations	19	Discuss limitations of the study, taking into account sources of potential bias or imprecision. Discuss both direction and magnitude of any potential bias	p13-14
Interpretation	20	Give a cautious overall interpretation of results considering objectives, limitations, multiplicity of analyses, results from similar studies, and other relevant evidence	p11-14
Generalisability	21	Discuss the generalisability (external validity) of the study results	p13-14
**Other information**			
Funding	22	Give the source of funding and the role of the funders for the present study and, if applicable, for the original study on which the present article is based	p14-15

*Give information separately for cases and controls in case-control studies and, if applicable, for exposed and unexposed groups in cohort and cross-sectional studies.

For peer review only

Note: An Explanation and Elaboration article discusses each checklist item and gives methodological background and published examples of transparent reporting. The STROBE checklist is best used in conjunction with this article (freely available on the Web sites of PLoS Medicine at http://www.plosmedicine.org/, Annals of Internal Medicine at http://www.annals.org/, and Epidemiology at http://www.epidem.com/). Information on the STROBE Initiative is available at www.strobe-statement.org.

For peer review only

Describing, predicting and explaining adherence to Total Skin Self-Examination (TSSE) in people with melanoma: a 12 month longitudinal study

Journal:	*BMJ Open*
Manuscript ID	bmjopen-2021-056755.R1
Article Type:	Original research
Date Submitted by the Author:	21-Apr-2022
Complete List of Authors:	Allan, Julia; University of Aberdeen, Aberdeen Health Psychology Group<br>Johnston, D; University of Aberdeen<br>Johnston, Marie; University of Aberdeen, Aberdeen Health Psychology Group<br>Murchie, Peter; University of Aberdeen, Division of Applied Health Science
**Primary Subject Heading**:	Oncology
Secondary Subject Heading:	Oncology
Keywords:	Dermatological tumours < ONCOLOGY, Adult oncology < ONCOLOGY, PREVENTIVE MEDICINE

SCHOLARONE™ Manuscripts

I, the Submitting Author has the right to grant and does grant on behalf of all authors of the Work (as defined in the below author licence), an exclusive licence and/or a non-exclusive licence for contributions from authors who are: i) UK Crown employees; ii) where BMJ has agreed a CC-BY licence shall apply, and/or iii) in accordance with the terms applicable for US Federal Government officers or employees acting as part of their official duties; on a worldwide, perpetual, irrevocable, royalty-free basis to BMJ Publishing Group Ltd ("BMJ") its licensees and where the relevant Journal is co-owned by BMJ to the co-owners of the Journal, to publish the Work in this journal and any other BMJ products and to exploit all rights, as set out in our licence.

The Submitting Author accepts and understands that any supply made under these terms is made by BMJ to the Submitting Author unless you are acting as an employee on behalf of your employer or a postgraduate student of an affiliated institution which is paying any applicable article publishing charge ("APC") for Open Access articles. Where the Submitting Author wishes to make the Work available on an Open Access basis (and intends to pay the relevant APC), the terms of reuse of such Open Access shall be governed by a Creative Commons licence – details of these licences and which Creative Commons licence will apply to this Work are set out in our licence referred to above.

Other than as permitted in any relevant BMJ Author's Self Archiving Policies, I confirm this Work has not been accepted for publication elsewhere, is not being considered for publication elsewhere and does not duplicate material already published. I confirm all authors consent to publication of this Work and authorise the granting of this licence.

For peer review only

Describing, predicting and explaining adherence to Total Skin Self-Examination (TSSE) in people with melanoma: a 12 month longitudinal study

Julia L. Allan[1*], j.allan@abdn.ac.uk

Derek W. Johnston[2], d.johnston@abdn.ac.uk

Marie Johnston[1], m.johnston@abdn.ac.uk

Peter Murchie[3], p.murchie@abdn.ac.uk

[1] Institute of Applied Health Sciences, University of Aberdeen, Aberdeen, UK

[2] School of Psychology, University of Aberdeen, Aberdeen, UK

[3] Centre for Academic Primary Care, University of Aberdeen, Aberdeen, UK

* Corresponding author. Institute of Applied Health Sciences, Health Sciences Building, University of Aberdeen, Foresterhill, Aberdeen. AB25 2ZD. +44(0)1224 438103

Keywords: melanoma; total skin self examination; adherence

Wordcount: 4,016

For peer review only

ABSTRACT

Objectives: To describe trajectories in melanoma survivors' adherence to monthly total skin self-examination (TSSE) over 12 months, and to investigate whether adherence trajectories can be predicted from demographic, cognitive or emotional factors at baseline.

Design: A longitudinal observational study nested within the intervention arm of the ASICA randomised controlled trial.

Setting: Follow up secondary care in Aberdeen and Cambridge UK.

Participants: n=104 adults (48M/56F; mean age 58.83 years, SD 13.47, range 28-85 years; mean SIMD score 8.03, SD 1.73, range 2-10) who had been treated for stage 0-IIC primary cutaneous melanoma in the preceding 60 months and were actively participating in the intervention arm of the ASICA trial.

Interventions: All participants were using the ASICA intervention – a tablet-based intervention designed to support monthly TSSE.

Primary and secondary outcome measures: The primary outcome was adherence to guideline recommended (monthly) TSSE over 12 months. This was determined from time-stamped TSSE data recorded by the ASICA intervention app.

Results: Latent growth mixture models identified 3 TSSE adherence trajectories (adherent - 41%; drop-off -35%; and non-adherent -24%). People who were non-adherent were less likely to intend to perform TSSE as recommended, intending to do it more frequently (OR=0 .21, (95% CI 0.06, 0.81, p=.023) and were more depressed (OR =1.31 (CI , 1.06, 1.61, p=.011) than people who were adherent. People whose adherence dropped-off over time had less well developed action plans (OR=0.78, CI 0.63,0.96, p=.016) and lower self-efficacy about TSSE (OR =0.92, CI 0.86,0.99, p=.028) than people who were adherent.

Conclusions: Adherence to monthly TSSE in people treated for melanoma can be differentiated into adherent, drop-off and non-adherent trajectories. Collecting information about intentions to engage in TSSE, depression, self-efficacy and/or action planning at outset may help to identify those who would benefit from additional intervention.

Trial registration: ClinicalTrials.gov Identifier: NCT03328247. Registered 01/11/2017.

For peer review only

ARTICLE SUMMARY

Strengths and limitations of this study

- A large sample of people treated for primary cutaneous melanoma were observed over 12 months to determine frequency and maintenance of adherence to recommended total skin self-examination (TSSE) over time
- TSSE adherence was objectively recorded via interactions with an app
- A 3-class latent growth mixture model produced adherence classifications that were highly satisfactory statistically and interpretable
- The sample showed limited variance in socioeconomic status, tending to be more affluent than the general population
- Quality of TSSE (i.e., thoroughness) was not a focus of this study and should be explored in future studies

For peer review only

INTRODUCTION

Melanoma is a relatively common skin cancer, with more than 16,000 new cases each year in the UK (1). Post-diagnosis, around 20% of melanoma patients will experience a recurrence (2) and 4-8% will develop a new primary tumour (3), most commonly within 5 years. As the majority of melanomas are readily visible on the skin's surface and show observable changes over time, clinical guidelines for the management of potential recurrence emphasise the importance of regular skin examination (4,5).

Gold standard total skin self-examination (TSSE) involves regular 'careful, deliberate and purposeful examination of the skin' (6), ideally monthly (7). It has been estimated that regular TSSE can reduce mortality from melanoma by as much as 63% (6) as TSSE allows patients to detect recurrences / new primaries between scheduled follow ups (8-12) when tumours are thinner / at an earlier stage (13,14).

Despite the clear benefits of TSSE, adherence amongst people with melanoma is typically suboptimal. Surveys suggest that around 12% of patients never engage in recommended TSSE (15) having never been directly advised to perform it (16) or advised to perform it but not shown how (17). When TSSE is performed, it is often insufficiently frequent: a recent systematic review of 30 studies demonstrated that only a minority of melanoma patients (16-24%) adhere to recommendations by performing TSSE monthly (18).

Interventions developed to support engagement in regular TSSE, have demonstrated some positive results. For example, in one randomised controlled trial with over 1,300 primary care patients, those randomised to an intervention that supported TSSE performed significantly more TSSEs at 2, 6 and 12 months than the control group (19). However, other studies have suggested that the beneficial effects of TSSE interventions may dissipate over time (20).

As sustained adherence to monthly TSSE is 'crucial' in melanoma care (21), it has been suggested that regular reminders and booster educational sessions should be included in interventions to try and minimise drop off in optimal TSSE over time (21). The same authors report that self-efficacy (i.e., confidence in ability to perform a TSSE) and intentions to perform a TSSE are two of the strongest psychosocial predictors of engagement in TSSE (22) and that as such, should be included in TSSE interventions. Specifically, they suggest that TSSE interventions should incorporate clear demonstrations of how to perform TSSE and concrete instructions about what to do if a suspicious change is detected (to enhance self-efficacy) and should include techniques such as implementation intentions (short specific, goal focused plans, 23) to help people convert their intentions into actions.

For peer review only - http://bmjopen.bmj.com/site/about/guidelines.xhtml

One intervention which includes all of the content suggested to optimise TSSE over time is ASICA (Achieving Self-Directed Integrated Cancer Aftercare), a digital intervention designed to support high-quality, regular TSSE in people with cutaneous melanoma (24,25). The details of intervention development are reported elsewhere (25) but in brief, ASICA is a tablet (Android) delivered behavioural intervention based on the Information-Motivation-Behaviour model (26) and including behaviour change techniques based on Control Theory (27) and Implementation Intentions (23) to support engagement in TSSE. The ASICA intervention prompts and supports TSSE in people with melanoma by; 1) motivating people to perform TSSE; 2) building confidence (self-efficacy) in the ability to perform TSSE; 3) prompting monthly TSSE; 4) supporting thorough TSSE with body area guides; 5) prompting planning of TSSE and 6) prompting timely action if skin changes are detected.

ASICA was recently evaluated in a randomised controlled trial over 12 months with 240 melanoma patients (28) to determine the intervention's effect on TSSE behaviour and psychological wellbeing. One objective of the trial was to collect detailed data on the frequency and maintenance of TSSE behaviour over time in order to investigate whether clinically relevant patterns in adherence behaviour can be detected and predicted early on, and whether this knowledge can be used to optimise future interventions.

The present study aims to answer two research questions;

1. *What* does adherence to TSSE over 12 months look like? Are there distinct patterns of non-adherence?

2. *Why* does optimal and suboptimal TSSE adherence occur? That is, do groups with suboptimal adherence differ from those with optimal adherence on demographic (age, gender, socioeconomic status, rurality), socio-cognitive (intentions, self-efficacy, action plans) and/or emotional (recurrence worries, anxiety, depression) factors?

METHODS

Design

For peer review only - http://bmjopen.bmj.com/site/about/guidelines.xhtml

A longitudinal study of objectively recorded TSSE adherence in people with melanoma over 12 months. The study was embedded within the intervention arm of the recently completed ASICA randomised controlled trial (25,28).

Ethics Approval

The project received full approval from the North of Scotland Research Ethics Committee on 28th April 2017 (17/NS/0040). Written informed consent was obtained from all study participants.

Patient and Public Involvement

A detailed pilot study was conducted during the development of the ASICA project to ascertain patients' priorities, experiences and preferences. Interviews were carried out with 19 potential recipients of the ASICA intervention, and these interviews informed the development of the study research questions and selection of outcome measures. Patients were not directly involved in the design of the study but did inform the design via participation in the pilot study interviews. The burden of the ASICA intervention was assessed by patients in a qualitative sub study. Two patient representatives sat on the trial steering group, feeding into plans for recruitment and dissemination. The results of the project will be disseminated to all participants (other than those who opted out) via a postal newsletter.

ASICA Trial and Intervention

The ASICA trial was a two-arm, multi centre randomised controlled trial designed to test the effects of the ASICA intervention on TSSE frequency and psychological wellbeing in adults treated for a stage 0-IIC (29) primary cutaneous melanoma. Participants were randomly allocated on a 1:1 basis to the ASICA intervention (intervention plus usual care) or control (usual care only) group. Intervention participants received the app-based ASICA intervention. The app included information about the importance of monthly TSSE, instructional videos demonstrating TSSE, a digital map of the patient's own skin, a checkbox list of body parts to check, prompts to plan TSSE and the capability to send photographs of suspicious skin lesions to a dermatology nurse practitioner for review. As part of the intervention, participants were prompted monthly (i.e., in line with guideline frequency) to conduct a TSSE.

For peer review only - http://bmjopen.bmj.com/site/about/guidelines.xhtml

For peer review only

Participants

Participants were n=104 adults (48M/56F; mean age 58.83 years, SD 13.47, range 28-85 years; mean Scottish Index of Multiple Deprivation [SIMD] score 8.03, SD 1.73, range 2-10) who had been treated for stage 0-IIC primary cutaneous melanoma in the preceding 60 months and who were actively engaged in the intervention arm of the ASICA trial between 2018 and 2020. Sample size for the ASICA trial (n=240 total; n=120 intervention group) was pragmatically determined based on the need to capture anticipated diversity in the primary outcomes (anxiety, depression, worry and quality of life) and the number of participants it was possible to recruit in the available time (25).

Measures

TSSE adherence. TSSE adherence was determined from data collected by the ASICA intervention app. After completing a practice, participants were prompted every 30 days for the next 12 months to complete and report a TSSE. Adherence to TSSE guidelines was determined by assessing via the time-stamped data collected by the ASICA app whether participants had completed at least one TSSE during each 30 day period following the receipt of a prompt (i.e., had engaged in TSSE at least monthly). Each period was coded 1 (at least one report received) or 0 (no report received), with possible scores ranging from 0 (totally non-adherent) to 13 (totally adherent, 1 practice + 12 monthly TSSEs).

Demographic, cognitive and emotional factors were measured in a baseline questionnaire administered at the start of the ASICA trial.

Demographic factors.

Age in years and gender were self-reported. Socioeconomic status was coded 1 (most deprived) to 10 (least deprived) by linking postcodes to indices of deprivation (Scottish Index of Multiple Deprivation; English indices of deprivation (30,31). Rurality/Urbanity was coded from 1 (most urban) to 10 (most rural) (32,33).

Cognitive factors.

Intention towards engaging in TSSE was assessed with the item "In the next 12 months, do you intend to check your skin for early signs of skin cancer? [yes/no]…. If yes, how many times? [insert number]". In order to map intentions onto guideline behaviour, this item was

scored categorically as <12 (intention below guideline), =12 (guideline intention) or >12 (intention above guideline).

Self-efficacy towards TSSE was assessed with 4 items rated on a 10-point scale from ‘not at all confident’ to ‘highly confident’: “How confident are you that……you can check your own skin correctly?; that you will find time in the next 12 months to check your own skin?; that you will remember to check your own skin at least once a month?; that if you find a spot or mole of concern that you will take appropriate action?”

Action Planning about TSSE was assessed with 2 items rated on a 5-point scale from ‘strongly agree to ‘strongly disagree’: “I have made plans about when to examine my own skin” and “I have made plans about where I will be when I examine my own skin”.

All of the items measuring cognitive factors were developed for the ASICA study (to relate specifically to the TSSE context) but were based on standard and valid methods of assessing the relevant theoretical constructs (34, 35, 36).

Emotional factors.

Anxiety and depression were measured using the 14 item Hospital Anxiety & Depression Scale (HADS; 37), a valid measure with adequate internal consistency (38). Anxiety and depression were scored separately (from 0-4), with higher scores indicating greater distress.

Melanoma specific worries were assessed with the 4 item Melanoma Worry Scale (39). Items were rated on a 5 point scale from ‘not at all’ to ‘almost all the time’ and assessed the frequency and impact of worries about possible recurrence.

Analysis

Latent group mixture modelling (LGMM) was carried out with Mplus V8.4 (40) to establish if there were systematically different classes of TSSE over the 12 month measurement period. A series of models were estimated from the TSSE adherence data with 1 to 5 latent classes, and were compared on the Bayesian information criterion, entropy values and number of participants per class. Class membership was obtained solely on the basis of the adherence data (i.e., covariates were not included in the modelling exercise), and the resulting classification information was then combined with the other information to explore the

For peer review only

For peer review only - http://bmjopen.bmj.com/site/about/guidelines.xhtml

differences between the classes using the classical three-step method (41). In the present analysis, the LGMM identified 3 classes of adherence behaviour (adherent, drop-off and non-adherent) by modelling the proportion of people who had completed the recommended monthly TSSE within each successive 30 day period over 12 months. Higher proportional estimates in this modelling exercise mean that more people are completing the recommended TSSE within each time window. Logistic regressions were then conducted using SPSS v25 to predict membership of these 3 classes from demographic (age, gender, socioeconomic status, rurality), cognitive (intentions, self-efficacy and action plans about TSSE) and emotional (anxiety, depression and melanoma worry) factors. In these logistic regression analyses, the dependent variable was dummy coded (with the adherent coded as 0 and the non-adherent or drop-off coded as 1). Categorical intention scores were treated as unordered categories. Missing data, which were <4% on all measures other than intention and 11.5% for intention, were dealt with by item deletion. Intention analyses were re-run with missing values imputed and results did not change.

RESULTS

What does adherence to TSSE over 12 months look like? Are there distinct patterns of non-adherence?

Of the 104 participants, 12 did not submit any TSSE reports using ASICA and the remainder submitted between 1 and 13 (median 6.5, IQR 9.75).

LGMM was tested with 1 to 5 latent groups. The lowest (best) Bayesian Information Criterion (BIC) was for a 3 class solution (1318). The entropy score (a measure of the quality of the classification) for the 3 class solution was also highly satisfactory (.906). The number of participants per class was substantial with the best fitting model classifying reasonable numbers (n=25/n=36/n=43) into categories that were readily interpretable (as showing consistent non-adherence; adherence which dropped off over time; and consistent adherence respectively). The three class solution was therefore selected for further study.

Figure 1 shows the LGMM estimated and observed values plotted over time and illustrates the 3 distinct classes identified. The largest class are people who are adherent (n=43) who

For peer review only - http://bmjopen.bmj.com/site/about/guidelines.xhtml

achieve over 90% adherence across the study period and show no significant decrease over time (p=.188). The next class, people whose adherence dropped-off over time (n=36), are those who start well, particularly after the first prompt but whose TSSE adherence declines steadily over 12 months (p< .001) until only around 20% are providing the required reports by the final month. The final group, people who are non-adherents (n=25) start poorly, submitting only 28% of reports in the first month, and decrease rapidly such that they are providing virtually no reports by 6 months (p=.022). An overview of the characteristics of participants in the three TSSE adherence classes can be found in Table 1. The accuracy of the classification (likely versus actual classification) was high (adherent =0.98; drop-off =0.93; non-adherent = 0.96).

Table 1: Demographic, cognitive and emotional factors in the three adherence groups

	Adherent	Drop-Off	Non-Adherent
N	43	36	25
Age in years (M, SD)	60.45 (11.77)	57.12 (13.31)	58.50 (16.38)
Gender (M/F)	16/27	20/16	12/13
Rurality (urban/rural)	21/22	23/13	18/7
Social Deprivation (M, SD)	7.98 (1.50)	8.17 (1.72)	7.92 (2.14)
Action Planning (M, SD)	6.86 (2.10)	5.56 (2.47)	6.25 (2.42)
Self-Efficacy (M, SD)	32.32 (5.89)	28.85 (7.04)	30.50 (6.90)
Intentions (n)*			
<12 times	13	15	6
=12 times	22	11	7
>12 times	6	3	9
HADS Anxiety (Mean, SD)	4.23 (3.99)	5.33 (3.84)	6.13 (4.95)
HADS Depression (Mean, SD)	1.93 (2.19)	3.14 (3.45)	3.75 (3.05)
Melanoma Worry (Mean, SD)	8.33 (3.52)	8.73 (3.59)	8.75 (3.90)

* Only 92/104 participants completed the intention measure

Why does optimal and suboptimal TSSE adherence occur? That it is, do groups with suboptimal adherence differ from those with optimal adherence on demographic (age,

For peer review only - http://bmjopen.bmj.com/site/about/guidelines.xhtml

gender, socioeconomic status, rurality) socio-cognitive (intentions, self-efficacy, action plans) and/or emotional (recurrence worries, anxiety, depression) factors?

The results of the univariate logistic regression analyses predicting adherence class (adherent; drop-off; non-adherent) from the demographic, cognitive and emotional measures are shown in Table 2 (comparison of non-adherent and adherent) and Table 3 (comparison of drop-offs and adherent). Supplementary multivariate analyses can be found in file S1.

Table 2: Logistic regression analyses of demographic, cognitive and emotional factors in people who are **non-adherent** (comparison group) **vs adherent.**

	B	SE	Wald	p	OR	95% CI OR
**Univariate**						
Age	0.01	0.02	0.33	0.566	0.99	[.95,1.03]
Gender	0.44	0.51	0.76	0.385	1.56	[.57,4.23]
SIMD	-0.02	0.14	0.02	0.9	0.98	[.74,1.30]
Rurality	-0.99	0.54	3.37	0.066	0.37	[.13,1.07]
Intention <12 **	-1.18	0.72	2.67	0.103	0.31	[.08,1.27]
Intention =12**	-1.55	0.68	5.17	0.023*	0.21	[.06,.81]
Action plan	-0.13	0.12	1.17	0.28	0.88	[.70,1.11]
Self-efficacy	-0.04	0.05	1.18	0.28	0.96	[.88,1.04]
HADS anxiety	0.1	0.06	2.76	0.096	1.10	[.98,1.24]
HADS depression	0.27	0.11	6.43	0.011*	1.31	[1.06,1.61]
MWS	0.04	0.07	0.23	0.633	1.04	[0.90,1.19]

* =significant at p<.05. ** Intention >12 used as comparison

Table 3: Logistic regression analyses of demographic, cognitive and emotional factors in people whose adherence **dropped-off** over time **(**comparison group) vs people who are adherent

	B	SE	Wald	p	OR	95% CI OR
**Univariate**						
Age	-0.02	0.02	1.38	0.241	0.98	[.94,1.02]
Gender	0.75	0.46	2.63	0.105	2.11	[.86,5.2]
SIMD	-0.08	0.14	0.28	0.597	1.08	[.81,1.43]
Rurality	-0.62	(0.46)	1.78	0.182	0.54	[.22,1.33]

For peer review only - http://bmjopen.bmj.com/site/about/guidelines.xhtml

Intention <12**	0.84	0.8	1.09	0.297	2.31	[.48,11.12]
Intention =12**	0	0.8	0	1	1	[.21,4.78]
Action plan	-0.25	0.11	5.77	0.016*	0.78	[.63,.96]
Self-efficacy	-0.08	0.04	4.82	0.028*	0.92	[.86,.99]
HADS Anxiety	0.07	0.06	1.52	0.217	1.08	[.96,1.21]
HADS Depression	0.16	0.09	3.19	0.074	1.17	[.98,1.40]
MWS	0.04	0.07	0.26	0.61	1.06	[.91,1.18]

* =significant at p<.05. ** Intention >12 used as comparison

For peer review only

Age, sex and SIMD did not significantly predict adherence class in any analysis, indicating that demographic factors alone are not sufficient to predict different adherence trajectories over time. However there was a tendency for rural participants to be more adherent.

In univariate comparisons of *cognitive factors*, people who were non-adherent were less likely to intend to carry out TSSE monthly than people who were adherent, instead reporting (potentially unrealistic) intentions to perform TSSE more often than monthly. People who were non–adherent did not differ from people who were adherent in terms of action plans or self-efficacy towards TSSE. People whose adherence dropped-off did not differ significantly from people who were adherent in their TSSE intentions. However the people whose adherence dropped-off reported lower levels of action planning and self-efficacy than people who were adherent. When *emotional factors* were compared, people who were non-adherent had significantly higher depression scores than people who were adherent with people whose adherence dropped off intermediate but not reliably different from people who were adherent. Anxiety was slightly higher in people who were non-adherent but this was not significant at p<.05. Melanoma worries did not significantly differ between any of the adherence groups.

DISCUSSION

Distinct patterns in TSSE adherence emerged in participants using the ASICA intervention over 12 months. Of the 104 participants, 41% (those in the 'adherent' category) consistently completed >90% of guideline recommended TSSEs over 12 months, which compares favourably to the 16-24% of participants reporting optimal TSSE adherence in a recent systematic review (18). However, even with the theory and evidence-based support offered by ASICA, the remaining 59% of participants in the present study showed one of two patterns of suboptimal adherence. Approximately 35% of people (those in the 'drop-off' category) initially adhered well but showed steady and significant declines in TSSE adherence over time until only around a fifth were conducting TSSEs by 12 months. The remaining 24% (those in the 'non-adherent' category) consistently failed to engage in monthly TSSEs, with only a minority (around a third) conducting a TSSE in the first month and virtually none completing one from 6 months onwards.

Predictors of Adherence

One of the aims of the present study was to determine whether participants who would go on to display suboptimal adherence could be identified at baseline so that they could be better supported from intervention outset. To this end, adherence trajectories were predicted from a range of easily measurable and theoretically relevant demographic, cognitive and emotional factors. None of the demographic factors assessed in the current study (age, gender, socioeconomic status, rurality) differentiated between classes of TSSE adherence behaviour, although there was a slight tendency for those living in urban areas to be less adherent. It should be noted however, that participants in this study showed extremely limited variance in socioeconomic status, tending to be more affluent than the general population. This restricted range likely reflects the combined effects of melanoma incidence being higher in more affluent individuals (e.g., 42), and trial participation being skewed towards individuals from higher socioeconomic groups, and limits the conclusions that can be drawn about socioeconomic status.

When cognitive factors (intentions about TSSE, self-efficacy towards TSSE and plans about when and where to perform TSSE) were examined, the data suggested that different cognitive processes may be involved in the two suboptimal patterns of adherence observed ('non-adherent' and 'drop-off'). People who were non-adherent were significantly more likely to have high and potentially unrealistic intentions about TSSE than people who were adherent: with many intending to perform TSSEs more frequently than monthly. Neither TSSE self-efficacy nor action planning significantly differentiated people who were non-

For peer review only - http://bmjopen.bmj.com/site/about/guidelines.xhtml

adherent from people who were adherent. In contrast, the opposite pattern was found to differentiate the people whose adherence dropped off from people who were adherent: the two groups did not significantly differ in their intentions about TSSE but people whose adherence dropped-off showed significantly lower levels of action planning and self-efficacy than people who were adherent. The observed pattern of results fits well with theoretical models of health behaviour change such as the Health Action Process Approach (HAPA; 43). HAPA explicitly distinguishes between motivational processes (such as intentions) involved in the early goal-setting phase of behaviour change, and volitional processes (such as self-efficacy and planning) involved in the later goal-pursuit phase of behaviour change. In the present study, the participants who were least likely to 'get started' (i.e., the people who were non-adherent) showed measurable differences in motivational processes (intentions) relative to people who were adherent. Similarly, those who started well (had motivation) but then had issues with maintaining the volitional aspects of regular TSSE over time (i.e., those whose adherence dropped-off) showed lower levels of self-efficacy and planning than those who were adherent. Cognitive factors were on average less predictive of TSSE behaviour in the present study than in other melanoma studies (22), possibly because the ASICA intervention was designed to boost intentions, self-efficacy and planning. This may have reduced natural variance in these constructs, limiting their predictive power.

The analysis of the emotional factors (anxiety, depression and melanoma specific worries) indicated that while people who were adherent consistently had the 'best' scores (i.e., lowest anxiety, lowest depression, fewest worries about recurrence), the only factor that significantly differentiated peoples who were adherent from people who were non-adherent was depression. People whose adherence dropped-off in contrast, did not significantly differ from people who were adherent on any of the emotional factors. It is noteworthy that melanoma specific worries were remarkably consistent across adherence groups and did not predict TSSE behaviour. Fears about recurrence are often suggested as a likely predictor of TSSE behaviour (i.e., that people worried about recurrence will check their skin more frequently, potentially more frequently than recommended), and recent evidence suggests that people who are more worried about recurrence are more likely to conduct TSSE and to conduct more thorough TSSE (44). The present results were not in line with this, suggesting that recurrence fears were unrelated to TSSE adherence over 12 months.

Clinical Implications

For peer review only

For peer review only - http://bmjopen.bmj.com/site/about/guidelines.xhtml

The present results suggest that routinely screening for fear of recurrence will not helpfully discriminate melanoma patients in terms of their likely future TSSE adherence. Making baseline assessments of participants' intentions about TSSE and level of depression however may serve to highlight the group most likely to be non-adherent. Intentions, if suboptimal, can in theory be strengthened with three behaviour change techniques: giving information about the health consequences of TSSE, setting goals about completing TSSE and providing an incentive for reaching the outcome (45). Similarly, depression might be reduced by helping patients to more effectively manage negative emotions, e.g., through stress management techniques (45). It is important to note however that the effect sizes in the present study were small so the potential clinical benefit of this approach would be limited.

Identifying those who start well but falter (those whose adherence 'drops-off') is challenging. While measures of self-efficacy and action planning differentiated people whose adherence dropped off from people who were adherent on average, they would not reliably identify people on this adherence trajectory at baseline (e.g., baseline self-efficacy scores in the present data set would correctly identify only 46% of drop-offs). Instead, adherence behaviour would have to be monitored for 6 months (in the present data), to identify 75% of people whose adherence dropped off (as those who have completed <4 of the recommended 6 TSSEs). Top up interventions could be targeted at this point: action planning might be addressed by prompting people to make 'if-then' plans (23) and self-efficacy might be enhanced by methods such as verbal persuasion and focusing on past success (e.g., in adhering in the early stages) (45). If top-up interventions were to be used, it would be possible in the present data to accurately identify 92% of people who were non-adherent and 100% of people who were adherent at 3 months on the basis of TSSEs completed (0-1 vs 2-3).

Strengths and Limitations

The present study has several methodological strengths. It uses objectively recorded TSSE behaviour rather than self-reports, and we can therefore be confident that TSSE took place at the times indicated. The study also followed participants over a full 12 months and tested both emotional and socio-cognitive models of behaviour. However, as discussed above, one notable limitation of the current study is that participants came almost exclusively from the less deprived end of the socioeconomic spectrum. While broadly representative of the clinical population (melanoma being more common in high SES individuals) the results reported are not representative of the general population. This raises an important point about whether

For peer review only - http://bmjopen.bmj.com/site/about/guidelines.xhtml

digital interventions like ASICA would exacerbate existing health inequalities if uptake (and therefore potential to benefit) is higher in individuals from less deprived sectors of the population (cf.46,47), or whether they would usefully ‘free up’ face to face resources for those least likely to engage with remote healthcare

Future Directions

Future studies should investigate in more detail methods for early detection of those least likely to be adherent. Based on our data, interventions which boost action planning and self-efficacy at 3 and 6 months might be beneficial in reducing the number people whose adherence drops-off while an intervention prior to starting monthly TSSE to address motivation and depression might reduce the number of people who go on to be non-adherent. Detection of sub-optimal adherence on an individual level would require real time measurement and more dynamic and individualised assessment in order to model the thresholds beyond which optimal adherence can be reliably distinguished from adherence which is likely to decline in future. Future studies should also extend the present work on the frequency and maintenance of TSSE to TSSE thoroughness. Studies which have investigated the thoroughness of TSSE, suggest that only 7-14% of patients check their whole body while performing TSSE (44,48) and only 9-13% use a mirror or ask another person to help them check areas that are difficult to see (48). Melanoma can recur anywhere on the body and more thorough SSE is associated with detection of earlier stage, more survivable lesions (49).

In conclusion, the present study demonstrates that adherence to recommended monthly TSSE in individuals treated for melanoma can be reliably differentiated into three distinct trajectories over time: adherent, drop-off and non-adherent. Collecting information about intentions to engage in TSSE, depression level, self-efficacy and/or action planning at outset may help to distinguish between those who go on to engage in optimal, declining or consistently suboptimal adherence.

Funding statement

This work was supported by a grant from a Cancer Research UK Population Research Committee project award (C10673/A21685). The views and opinions expressed herein are those of the authors and do not necessarily reflect those of Cancer Research UK. The funder (through their peer review and funding board review process) approved the study proposal but had no role in the collection, analysis, or interpretation of data, or writing of the report.

For peer review only - http://bmjopen.bmj.com/site/about/guidelines.xhtml

Competing interests statement

None of the authors have competing interests to declare.

Author contributions

JA, MJ, DWJ and PM conceived of, designed and planned the study. DWJ analysed the data in consultation with JA, MJ and PM. JA drafted the manuscript. JA, DWJ, MJ and PM interpreted the results and revised the manuscript. JA is responsible for the overall content.

Data sharing statement

Data are available on request.

Figure Legend

Figure 1: The 3 classes of adherence to TSSE over time showing LGMM estimated (statistically predicted) and observed (actual) proportion of participants completing TSSE within each 30 day period.

References

1. Cancer Research UK. Melanoma skin cancer statistics 2015-17. Retrieved from https://www.cancerresearchuk.org/health-professional/cancer-statistics/statistics-by-cancer-type/melanoma-skin-cancer

2. Francken AB, Accortt NA, Shaw HM, *et al*. Follow-up schedules after treatment for malignant melanoma. *Br J Surg* 2008;95:1401-1407. https://doi.org/10.1002/bjs.6347

3. Di Fronzo LA, Wanek LA, Elashoff R *et al*. Increased incidence of second primary melanoma in patients with a previous cutaneous melanoma. *Ann Surg Oncol* 1999;6:705711.

4. National Institute for Health and Care Excellence (NICE). Melanoma: Assessment and management (NG14; 2015). Retrieved from https://www.nice.org.uk/guidance/ng14

5. Health Improvement Scotland. SIGN QRG 146 Cutaneous Melanoma. 2016. Retrieved from https://www.sign.ac.uk/assets/qrg146.pdf

6. Berwick M, Begg CB, Fine JA. Screening for cutaneous melanoma by skin self-examination. *J Natl Cancer Inst* 1996;88:17–23.

7. Watts CG, Dieng M, Morton RL, *et al*. Clinical practice guidelines for identification, screening and follow-up of individuals at high risk of primary cutaneous melanoma: a systematic review. *Br J Dermatol* 2015;172:33–47. https://doi.org/10.1111/bjd.13403.

For peer review only - http://bmjopen.bmj.com/site/about/guidelines.xhtml

8. Dicker TJ, Kavanagh GM, Herd RM, *et al*. A rational approach to melanoma follow-up in patients with primary cutaneous melanoma. Scottish Melanoma Group. *Br J Dermatol* 1999;140:249–254. https://doi.org/10.1046/j.1365-2133.1999.02657.x

9. Meyers MO, Yeh JJ, Frank J, *et al* (2009). Method of detection of initial recurrence of stage II/III cutaneous melanoma: analysis of the utility of follow-up staging. *Ann Surg Oncol*, 2009;16:941–947. https://doi.org/10.1245/s10434-008-0238-y

10. Auckland R, Wassell P, Hall S, *et al*. Exploring patterns of recurrent melanoma in Northeast Scotland to inform the introduction a digital self-examination intervention. *BMC Dermatol* 2014;14:4. https://doi.org/10.1186/1471-5945-14-4

11. Brady MS, Oliveria SA, Christos PJ, *et al*. Patterns of detection in patients with cutaneous melanoma. *Cancer* 2000;89:342–347.

12. Swetter SM, Pollitt RA, Johnson TM, *et al*. Behavioral determinants of successful early melanoma detection: role of self and physician skin examination. *Cancer* 2012;118:3725–34. https://doi.org/10.1002/cncr.26707.

13. Moore Dalal K, Zhou Q, Panageas KS, *et al*. Methods of detection of first recurrence in patients with stage I/II primary cutaneous melanoma after sentinel lymph node biopsy. *Ann Surg Oncol* 2008;15:2206-2214. doi: 10.1245/s10434-008-9985-z

14. Paddock LE, Lu SE, Bandera EV, *et al*. Skin self-examination and long-term melanoma survival. *Melanoma Res* 2016;26:401–408.

15. Hull PR, Piemontesi NG, Lichtenwald J. Compliance with self-examination surveillance in patients with melanoma and atypical moles: an anonymous questionnaire study. *J Cutan Med and Surg* 2011;15:97–102. https://doi.org/10.2310/7750.2011.10011

16. Körner A, Coroiu A, Martins C, *et al*. Predictors of skin self-examination before and after a melanoma diagnosis: the role of medical advice and patient's level of education. *Int Arch Med* 2013;6:8. https://doi.org/10.1186/1755-7682-6-8

17. Manne S, Lessin S. Prevalence and Correlates of Sun Protection and Skin Self-Examination Practices Among Cutaneous Malignant Melanoma Survivors. *J Behav Med* 2006;29:419–434. https://doi.org/10.1007/s10865-006-9064-5

18. Bhurosy T, Heckman CJ, Riley M. Prevalence and correlates of skin self-examination behaviors among melanoma survivors: a systematic review, *Transl Behav Med* 2020;10:1120–1133, https://doi.org/10.1093/tbm/ibaa003

19. Weinstock MA, Risica PM, Martin RA, *et al*. Melanoma early detection with thorough skin self-examination: the "Check It Out" randomized trial. *Am J Prev Med* 2007; 32:517–524. https://doi.org/10.1016/j.amepre.2007.02.024

For peer review only - http://bmjopen.bmj.com/site/about/guidelines.xhtml

20. Janda M, Neale RE, Youl P, *et al*. Impact of a video-based intervention to improve the prevalence of skin self-examination in men 50 years or older: the randomized skin awareness trial. *Arch Dermatol* 2011;147:799–806. https://doi.org/10.1001

21. Coroiu A, Moran C, Bergeron C, *et al.* Short and long-term barriers and facilitators of skin self-examination among individuals diagnosed with melanoma. *BMC Cancer* 20**;**123. https://doi.org/10.1186/s12885-019-6476-5

22. Coroiu A, Moran C, Bergeron C, *et al* (2020a). Operationalization of skin self-examination in randomized controlled trials with individuals at increased risk for melanoma: A systematic review. *Pat Ed Couns*, 2020;*103*:1013–1026. https://doi.org/10.1016/j.pec.2019.12.009

23. Gollwitzer PM. Implementation intentions: strong effects of simple plans. *Am Psychol* 1999;54:493.

24. Murchie P, Allan JL, Brant W, *et al*. Total skin self-examination at home for people treated for cutaneous melanoma: development and pilot of a digital intervention. *BMJ Open* 2015;5:e007993

25. Murchie P, Masthoff J, Walter FM, *et al.* Achieving Self-Directed Integrated Cancer Aftercare (ASICA) in melanoma: protocol for a randomised patient-focused pilot trial of delivering the ASICA intervention as a means to earlier detection of recurrent and second primary melanoma. *Trials* 2019;20**:**318. https://doi.org/10.1186/s13063-019-3453-x

26. Fisher JD, Fisher WA. Changing AIDS-risk behavior. *Psychol Bull* 1992;111:455–74

27. Carver CS, Scheier MF. Attention and self-regulation: a control theory approach to human behaviour. New York: Springer; 1981.

28. Murchie P, Constable L, Hall S, *et al*. A randomised patient-focused trial of the Achieving Self-Directed Integrated Cancer Aftercare (ASICA) intervention for detection of recurrent and second primary melanoma: effect on psychological well-being and quality of life. In submission.

29. Keung EZ, Gershenwald JE. The eighth edition American Joint Committee on Cancer (AJCC) melanoma staging system: implications for melanoma treatment and care. *Expert Rev Anticancer Ther*, 2018;18:775–784. https://doi.org/10.1080/14737140.2018.1489246

30. Scottish Government. Scottish Index of Multiple Deprivation. 2020. Retrieved from https://www.gov.scot/collections/scottish-index-of-multiple-deprivation-2020/

31. UK Government. National Statistics English indices of deprivation 2019. Retrieved from https://www.gov.uk/government/statistics/english-indices-of-deprivation-2019

32. Scottish Government. Urban Rural Classification 2016. Retrieved from https://www.gov.scot/publications/scottish-government-urban-rural-classification-2016/pages/2/

For peer review only

33. Office for National Statistics. Rural/urban classification 2011. Retrieved from https://www.ons.gov.uk/methodology/geography/geographicalproducts/ruralurbanclassifications

34. Francis J, Eccles MP, Johnston M, Walker AE, Grimshaw JM, Foy R, Kaner EFS, Smith L, Bonetti D. Constructing questionnaires based on the theory of planned behaviour: A manual for health services researchers. Newcastle upon Tyne, UK 2004: Centre for Health Services Research, University of Newcastle upon Tyne.

35. Burrell AMG, Allan JL, Williams DM, Johnston M. What do self-efficacy items measure? Examining the discriminant content validity of self-efficacy items. *Br J Health Psychol*, 2018;23:597-611.

36. Sniehotta FF, Scholz U, Schwarzer R. Bridging the intention–behaviour gap: Planning, self-efficacy, and action control in the adoption and maintenance of physical exercise, *Psychol Health* 2005;20:143-160

37. Zigmond AS, Snaith RP. The hospital anxiety and depression scale. *Acta Psychiat Scand* 1983;67:361-70.

38. Bjelland I, Dahl AA, Haug TT, et al. The validity of the Hospital Anxiety and Depression Scale: An updated literature review. *J Psychosom Res* 2002;52:69-77.

39. Moye MS, King SMC, Rice ZP, *et al*. Effects of total-body digital photography on cancer worry in patients with atypical mole syndrome. *JAMA Dermatol*, 2015;151:137–43. https://doi.org/10.1001/jamadermatol.2014.2229

40. Muthen LK, Muthen BO. Mplus User Guide. Eighth Edition. 1988-2017. Los Angeles, CA: Muthen & Muthen.

41. Wang J, Wang X. Structural Equation Modeling: Applications Using Mplus. Second Edition. 2020. John Wiley & Sons.

42. Johnson-Obaseki SE, Labajian V, Corsten MJ, *et al*. Incidence of cutaneous malignant melanoma by socioeconomic status in Canada: 1992-2006. *J Otolaryng Head Neck Surg* 2015;*44*:53. https://doi.org/10.1186/s40463-015-0107-1

43. Schwarzer R. Self-efficacy in the adoption and maintenance of health behaviours: Theoretical approaches and a new model. In: Schwarzer R, editor. Self-Efficacy: Thought Control of Action. Washington: Taylor and Francis; 1992. pp. 217–243.

44. Manne SL, Heckman CJ, Kashy D, *et al*. Prevalence and correlates of skin self-examination practices among cutaneous malignant melanoma survivors. *Prev Med Rep* 2020;19. https://doi.org/10.1016/j.pmedr.2020.101110.

45. Johnston M, Carey RN, Connell LE, *et al*. Development of an online tool for linking behavior change techniques and mechanisms of action based on triangulation of findings from literature synthesis and expert consensus. *Translat Behav Med* 2021;5:1049-1065. https://doi.org/10.1093/tbm/ibaa050

For peer review only

For peer review only - http://bmjopen.bmj.com/site/about/guidelines.xhtml

46. McAuley A. Digital health interventions: widening access or widening inequalities? *Pub Health* 2014;12:1118-1120.

47. Honeyman M, Maguire D, Evans H, *et al*. Digital technology and health inequalities: a scoping review. 2020. Cardiff: Public Health Wales NHS Trust.

48. Coups EJ, Manne SL, Stapleton JL, *et al*. Skin self-examination behaviors among individuals diagnosed with melanoma. *Melanoma Res* 2016;26:71–6. https://doi.org/10.1097/CMR.0000000000000204.

49. Pollitt RA, Geller AC, Brooks DR, *et al*. Efficacy of skin self-examination practices for early melanoma detection. *Cancer Epidemiol Biomark Prev* 2009;18:3018–23. https://doi.org/10.1158/1055-9965.epi-09-0310.

For peer review only

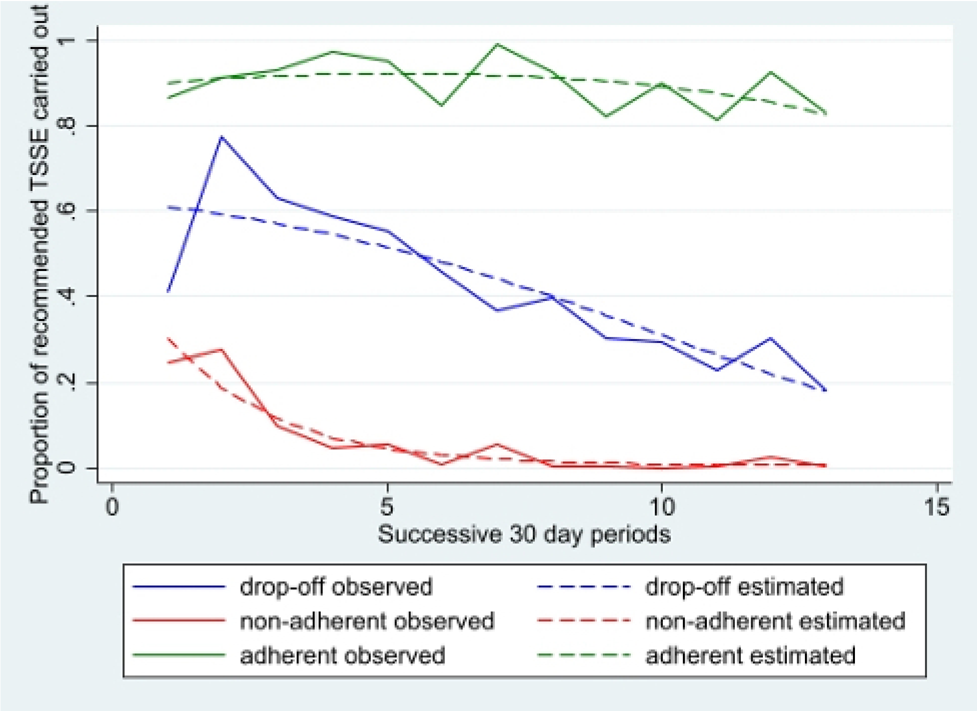

Figure 1: The 3 classes of adherence to TSSE over time showing LGMM estimated and observed values

172x125mm (72 x 72 DPI)

Supplementary File S1

Multivariate analyses

The main analyses reported in the manuscript are univariate logistic regressions. As a supplementary analysis, any predictors that were significant at p<.1 were entered into further multivariate analyses. These multivariate analyses are shown below in Tables S1 and S2.

When intention, anxiety and depression (i.e. all predictors where p<.1) were included in the analysis of non-adherent versus adherent participants, intentions about TSSE and depression remained significant, each explaining unique variance. People who were non-adherent were more depressed and more likely to intend to conduct TSSE more frequently than recommended than people who were adherent.

When action planning and self-efficacy (as predictors where p<.1) were included in a multivariate analysis of the drop offs versus the adherent participants, neither was significant. This may reflect the fact that efficacy is a theoretical precursor of action planning, and as such, the two will be highly inter-related and may explain the same variance.

Table S1: Multivariate logistic regression analyses of demographic, cognitive and emotional factors in people who are **non-adherent** (comparison group) **vs adherent.**

	B	SE	Wald	p	OR	95% CI OR
**Multivariate**						
Rurality	-0.91	0.64	2.05	0.152	0.40	[.12,1.40]
Intention<12	-1.69	0.84	4.09	0.043*	0.18	[.04,.95]
Intention=12	-1.70	0.74	5.31	0.021*	0.18	[.04,.78]
HADS Anxiety	-0.01	0.09	0.03	0.87	0.99	[.83,1.17]
HADS Depression	0.34	0.15	4.91	0.027*	1.40	[1.04,1.89]

* =significant at p<.05. ** Intention >12 used as comparison

For peer review only

Table S2: Multivariate logistic regression analyses of demographic, cognitive and emotional factors in people whose adherence **dropped-off** over time (comparison group) vs people who are adherent

	B	SE	Wald	p	OR	95% CI OR
**Multivariate**						
Action Plan	-0.14	0.13	1.13	0.289	0.87	[.68,1.12]
Self-efficacy	-0.05	0.05	1.47	0.225	0.95	[.87,1.03]
HADS depression	0.14	0.09	2.28	0.132	1.15	[.96,1.38]

* =significant at p<.05. ** Intention >12 used as comparison

For peer review only

STROBE 2007 (v4) checklist of items to be included in reports of observational studies in epidemiology*
Checklist for cohort, case-control, and cross-sectional studies (combined)

Section/Topic	Item #	Recommendation	Reported on page #
**Title and abstract**	1	(*a*) Indicate the study's design with a commonly used term in the title or the abstract	p1-2
		(*b*) Provide in the abstract an informative and balanced summary of what was done and what was found	p2
**Introduction**			
Background/rationale	2	Explain the scientific background and rationale for the investigation being reported	p4-5
Objectives	3	State specific objectives, including any pre-specified hypotheses	p5
**Methods**			
Study design	4	Present key elements of study design early in the paper	p5-6
Setting	5	Describe the setting, locations, and relevant dates, including periods of recruitment, exposure, follow-up, and data collection	p6-7
Participants	6	(*a*) *Cohort study*—Give the eligibility criteria, and the sources and methods of selection of participants. Describe methods of follow-up<br>*Case-control study*—Give the eligibility criteria, and the sources and methods of case ascertainment and control selection. Give the rationale for the choice of cases and controls<br>*Cross-sectional study*—Give the eligibility criteria, and the sources and methods of selection of participants	p7
		(*b*) *Cohort study*—For matched studies, give matching criteria and number of exposed and unexposed<br>*Case-control study*—For matched studies, give matching criteria and the number of controls per case	N/A
Variables	7	Clearly define all outcomes, exposures, predictors, potential confounders, and effect modifiers. Give diagnostic criteria, if applicable	p7-8
Data sources/ measurement	8*	For each variable of interest, give sources of data and details of methods of assessment (measurement). Describe comparability of assessment methods if there is more than one group	p7-8
Bias	9	Describe any efforts to address potential sources of bias	p7-9
Study size	10	Explain how the study size was arrived at	p7
Quantitative variables	11	Explain how quantitative variables were handled in the analyses. If applicable, describe which groupings were chosen and why	p8-9
Statistical methods	12	(*a*) Describe all statistical methods, including those used to control for confounding	p8-9
		(*b*) Describe any methods used to examine subgroups and interactions	p8-9
		(*c*) Explain how missing data were addressed	p9
		(*d*) *Cohort study*—If applicable, explain how loss to follow-up was addressed<br>*Case-control study*—If applicable, explain how matching of cases and controls was addressed	N/A

For peer review only

		Cross-sectional study—If applicable, describe analytical methods taking account of sampling strategy	
		(*e*) Describe any sensitivity analyses	p9
**Results**			
Participants	13*	(a) Report numbers of individuals at each stage of study—eg numbers potentially eligible, examined for eligibility, confirmed eligible, included in the study, completing follow-up, and analysed	Table 1
		(b) Give reasons for non-participation at each stage	N/A
		(c) Consider use of a flow diagram	N/A
Descriptive data	14*	(a) Give characteristics of study participants (eg demographic, clinical, social) and information on exposures and potential confounders	Table 1
		(b) Indicate number of participants with missing data for each variable of interest	p9, Table 1
		(c) *Cohort study*—Summarise follow-up time (eg, average and total amount)	p7
Outcome data	15*	*Cohort study*—Report numbers of outcome events or summary measures over time	p9
		Case-control study—Report numbers in each exposure category, or summary measures of exposure	
		Cross-sectional study—Report numbers of outcome events or summary measures	
Main results	16	(*a*) Give unadjusted estimates and, if applicable, confounder-adjusted estimates and their precision (eg, 95% confidence interval). Make clear which confounders were adjusted for and why they were included	Tables 2 and 3
		(*b*) Report category boundaries when continuous variables were categorized	p8-9
		(*c*) If relevant, consider translating estimates of relative risk into absolute risk for a meaningful time period	N/A
Other analyses	17	Report other analyses done—eg analyses of subgroups and interactions, and sensitivity analyses	p7
**Discussion**			
Key results	18	Summarise key results with reference to study objectives	p9-12
Limitations	19	Discuss limitations of the study, taking into account sources of potential bias or imprecision. Discuss both direction and magnitude of any potential bias	p15-16
Interpretation	20	Give a cautious overall interpretation of results considering objectives, limitations, multiplicity of analyses, results from similar studies, and other relevant evidence	p13-15
Generalisability	21	Discuss the generalisability (external validity) of the study results	p15-16
**Other information**			
Funding	22	Give the source of funding and the role of the funders for the present study and, if applicable, for the original study on which the present article is based	p16

*Give information separately for cases and controls in case-control studies and, if applicable, for exposed and unexposed groups in cohort and cross-sectional studies.
Note: An Explanation and Elaboration article discusses each checklist item and gives methodological background and published examples of transparent reporting. The STROBE checklist is best used in conjunction with this article (freely available on the Web sites of PLoS Medicine at http://www.plosmedicine.org/, Annals of Internal Medicine at http://www.annals.org/, and Epidemiology at http://www.epidem.com/). Information on the STROBE Initiative is available at www.strobe-statement.org.

For peer review only

BMJ Open

Describing, predicting and explaining adherence to Total Skin Self-Examination (TSSE) in people with melanoma: a 12 month longitudinal study

Journal:	*BMJ Open*
Manuscript ID	bmjopen-2021-056755.R2
Article Type:	Original research
Date Submitted by the Author:	09-Aug-2022
Complete List of Authors:	Allan, Julia; University of Aberdeen, Aberdeen Health Psychology Group<br>Johnston, D; University of Aberdeen, Psychology<br>Johnston, Marie; University of Aberdeen, Aberdeen Health Psychology Group<br>Murchie, Peter; University of Aberdeen, Division of Applied Health Science
**Primary Subject Heading**:	Oncology
Secondary Subject Heading:	Oncology
Keywords:	Dermatological tumours < ONCOLOGY, Adult oncology < ONCOLOGY, PREVENTIVE MEDICINE

SCHOLARONE™
Manuscripts

I, the Submitting Author has the right to grant and does grant on behalf of all authors of the Work (as defined in the below author licence), an exclusive licence and/or a non-exclusive licence for contributions from authors who are: i) UK Crown employees; ii) where BMJ has agreed a CC-BY licence shall apply, and/or iii) in accordance with the terms applicable for US Federal Government officers or employees acting as part of their official duties; on a worldwide, perpetual, irrevocable, royalty-free basis to BMJ Publishing Group Ltd ("BMJ") its licensees and where the relevant Journal is co-owned by BMJ to the co-owners of the Journal, to publish the Work in this journal and any other BMJ products and to exploit all rights, as set out in our licence.

The Submitting Author accepts and understands that any supply made under these terms is made by BMJ to the Submitting Author unless you are acting as an employee on behalf of your employer or a postgraduate student of an affiliated institution which is paying any applicable article publishing charge ("APC") for Open Access articles. Where the Submitting Author wishes to make the Work available on an Open Access basis (and intends to pay the relevant APC), the terms of reuse of such Open Access shall be governed by a Creative Commons licence – details of these licences and which Creative Commons licence will apply to this Work are set out in our licence referred to above.

Other than as permitted in any relevant BMJ Author's Self Archiving Policies, I confirm this Work has not been accepted for publication elsewhere, is not being considered for publication elsewhere and does not duplicate material already published. I confirm all authors consent to publication of this Work and authorise the granting of this licence.

For peer review only

Describing, predicting and explaining adherence to Total Skin Self-Examination (TSSE) in people with melanoma: a 12 month longitudinal study

Julia L. Allan[1*], j.allan@abdn.ac.uk
D. Johnston[2], d.johnston@abdn.ac.uk
Marie Johnston[1], m.johnston@abdn.ac.uk
Peter Murchie[3], p.murchie@abdn.ac.uk

[1] Institute of Applied Health Sciences, University of Aberdeen, Aberdeen, UK
[2] School of Psychology, University of Aberdeen, Aberdeen, UK
[3] Centre for Academic Primary Care, University of Aberdeen, Aberdeen, UK

* Corresponding author. Institute of Applied Health Sciences, Health Sciences Building, University of Aberdeen, Foresterhill, Aberdeen. AB25 2ZD. +44(0)1224 438103

Keywords: melanoma; total skin self examination; adherence

Wordcount: 4,014

For peer review only

ABSTRACT

Objectives: To describe trajectories in melanoma survivors' adherence to monthly total skin self-examination (TSSE) over 12 months, and to investigate whether adherence trajectories can be predicted from demographic, cognitive or emotional factors at baseline.

Design: A longitudinal observational study nested within the intervention arm of the ASICA randomised controlled trial.

Setting: Follow up secondary care in Aberdeen and Cambridge UK.

Participants: n=104 adults (48M/56F; mean age 58.83 years, SD 13.47, range 28-85 years; mean SIMD score 8.03, SD 1.73, range 2-10) who had been treated for stage 0-IIC primary cutaneous melanoma in the preceding 60 months and were actively participating in the intervention arm of the ASICA trial.

Interventions: All participants were using the ASICA intervention – a tablet-based intervention designed to support monthly TSSE.

Primary and secondary outcome measures: The primary outcome was adherence to guideline recommended (monthly) TSSE over 12 months. This was determined from time-stamped TSSE data recorded by the ASICA intervention app.

Results: Latent growth mixture models identified 3 TSSE adherence trajectories (adherent - 41%; drop-off -35%; and non-adherent -24%). People who were non-adherent were less likely to intend to perform TSSE as recommended, intending to do it more frequently (OR=0 .21, (95% CI 0.06, 0.81, p=.023) and were more depressed (OR =1.31 (CI , 1.06, 1.61, p=.011) than people who were adherent. People whose adherence dropped-off over time had less well developed action plans (OR=0.78, CI 0.63,0.96, p=.016) and lower self-efficacy about TSSE (OR =0.92, CI 0.86,0.99, p=.028) than people who were adherent.

Conclusions: Adherence to monthly TSSE in people treated for melanoma can be differentiated into adherent, drop-off and non-adherent trajectories. Collecting information about intentions to engage in TSSE, depression, self-efficacy and/or action planning at outset may help to identify those who would benefit from additional intervention.

Trial registration: ClinicalTrials.gov Identifier: NCT03328247. Registered 01/11/2017.

For peer review only - http://bmjopen.bmj.com/site/about/guidelines.xhtml

ARTICLE SUMMARY

Strengths and limitations of this study

- A large sample of people treated for primary cutaneous melanoma were observed over 12 months to determine frequency and maintenance of adherence to recommended total skin self-examination (TSSE) over time
- TSSE adherence was objectively recorded via interactions with an app
- A 3-class latent growth mixture model produced adherence classifications that were highly satisfactory statistically and interpretable
- The sample showed limited variance in socioeconomic status, tending to be more affluent than the general population
- Quality of TSSE (i.e., thoroughness) was not a focus of this study and should be explored in future studies

For peer review only

INTRODUCTION

Melanoma is a relatively common skin cancer, with more than 16,000 new cases each year in the UK (1). Post-diagnosis, around 20% of melanoma patients will experience a recurrence (2) and 4-8% will develop a new primary tumour (3), most commonly within 5 years. As the majority of melanomas are readily visible on the skin's surface and show observable changes over time, clinical guidelines for the management of potential recurrence emphasise the importance of regular skin examination (4,5).

Gold standard total skin self-examination (TSSE) involves regular 'careful, deliberate and purposeful examination of the skin' (6), ideally monthly (7). It has been estimated that regular TSSE can reduce mortality from melanoma by as much as 63% (6) as TSSE allows patients to detect recurrences / new primaries between scheduled follow ups (8-12) when tumours are thinner / at an earlier stage (13,14).

Despite the clear benefits of TSSE, adherence amongst people with melanoma is typically suboptimal. Surveys suggest that around 12% of patients never engage in recommended TSSE (15) having never been directly advised to perform it (16) or advised to perform it but not shown how (17). When TSSE is performed, it is often insufficiently frequent: a recent systematic review of 30 studies demonstrated that only a minority of melanoma patients (16-24%) adhere to recommendations by performing TSSE monthly (18).

Interventions developed to support engagement in regular TSSE, have demonstrated some positive results. For example, in one randomised controlled trial with over 1,300 primary care patients, those randomised to an intervention that supported TSSE performed significantly more TSSEs at 2, 6 and 12 months than the control group (19). However, other studies have suggested that the beneficial effects of TSSE interventions may dissipate over time (20).

As sustained adherence to monthly TSSE is 'crucial' in melanoma care (21), it has been suggested that regular reminders and booster educational sessions should be included in interventions to try and minimise drop off in optimal TSSE over time (21). The same authors report that self-efficacy (i.e., confidence in ability to perform a TSSE) and intentions to perform a TSSE are two of the strongest psychosocial predictors of engagement in TSSE (22) and that as such, should be included in TSSE interventions. Specifically, they suggest that TSSE interventions should incorporate clear demonstrations of how to perform TSSE and concrete instructions about what to do if a suspicious change is detected (to enhance self-efficacy) and should include techniques such as implementation intentions (short specific, goal focused plans, 23) to help people convert their intentions into actions.

For peer review only - http://bmjopen.bmj.com/site/about/guidelines.xhtml

One intervention which includes all of the content suggested to optimise TSSE over time is ASICA (Achieving Self-Directed Integrated Cancer Aftercare), a digital intervention designed to support high-quality, regular TSSE in people with cutaneous melanoma (24,25). The details of intervention development are reported elsewhere (25) but in brief, ASICA is a tablet (Android) delivered behavioural intervention based on the Information-Motivation-Behaviour model (26) and including behaviour change techniques based on Control Theory (27) and Implementation Intentions (23) to support engagement in TSSE. The ASICA intervention prompts and supports TSSE in people with melanoma by; 1) motivating people to perform TSSE; 2) building confidence (self-efficacy) in the ability to perform TSSE; 3) prompting monthly TSSE; 4) supporting thorough TSSE with body area guides; 5) prompting planning of TSSE and 6) prompting timely action if skin changes are detected.

ASICA was recently evaluated in a randomised controlled trial over 12 months with 240 melanoma patients (28) to determine the intervention's effect on TSSE behaviour and psychological wellbeing. One objective of the trial was to collect detailed data on the frequency and maintenance of TSSE behaviour over time in order to investigate whether clinically relevant patterns in adherence behaviour can be detected and predicted early on, and whether this knowledge can be used to optimise future interventions.

The present study aims to answer two research questions;

1. What does adherence to TSSE over 12 months look like? Are there distinct patterns of non-adherence?

2. What factors predict suboptimal TSSE adherence? That is, do groups with suboptimal adherence differ from those with optimal adherence on demographic (age, gender, socioeconomic status, rurality), socio-cognitive (intentions, self-efficacy, action plans) and/or emotional (recurrence worries, anxiety, depression) factors?

METHODS

Design

For peer review only - http://bmjopen.bmj.com/site/about/guidelines.xhtml

A longitudinal study of objectively recorded TSSE adherence in people with melanoma over 12 months. The study was embedded within the intervention arm of the recently completed ASICA randomised controlled trial (25,28).

.

Patient and Public Involvement

A detailed pilot study was conducted during the development of the ASICA project to ascertain patients' priorities, experiences and preferences. Interviews were carried out with 19 potential recipients of the ASICA intervention, and these interviews informed the development of the study research questions and selection of outcome measures. Patients were not directly involved in the design of the study but did inform the design via participation in the pilot study interviews. The burden of the ASICA intervention was assessed by patients in a qualitative sub study. Two patient representatives sat on the trial steering group, feeding into plans for recruitment and dissemination. The results of the project will be disseminated to all participants (other than those who opted out) via a postal newsletter.

ASICA Trial and Intervention

The ASICA trial was a two-arm, multi centre randomised controlled trial designed to test the effects of the ASICA intervention on TSSE frequency and psychological wellbeing in adults treated for a stage 0-IIC (29) primary cutaneous melanoma. Participants were randomly allocated on a 1:1 basis to the ASICA intervention (intervention plus usual care) or control (usual care only) group. Intervention participants received the app-based ASICA intervention. The app included information about the importance of monthly TSSE, instructional videos demonstrating TSSE, a digital map of the patient's own skin, a checkbox list of body parts to check, prompts to plan TSSE and the capability to send photographs of suspicious skin lesions to a dermatology nurse practitioner for review. As part of the intervention, participants were prompted monthly (i.e., in line with guideline frequency) to conduct a TSSE.

Participants

Participants were n=104 adults (48M/56F; mean age 58.83 years, SD 13.47, range 28-85 years; mean Scottish Index of Multiple Deprivation [SIMD] score 8.03, SD 1.73, range 2-10) who had been treated for stage 0-IIC primary cutaneous melanoma in the preceding 60

For peer review only

months and who were actively engaged in the intervention arm of the ASICA trial between 2018 and 2020. Sample size for the ASICA trial (n=240 total; n=120 intervention group) was pragmatically determined based on the need to capture anticipated diversity in the primary outcomes (anxiety, depression, worry and quality of life) and the number of participants it was possible to recruit in the available time (25).

Measures

TSSE adherence. TSSE adherence was determined from data collected by the ASICA intervention app. After completing a practice, participants were prompted every 30 days for the next 12 months to complete and report a TSSE. Adherence to TSSE guidelines was determined by assessing via the time-stamped data collected by the ASICA app whether participants had completed at least one TSSE during each 30 day period following the receipt of a prompt (i.e., had engaged in TSSE at least monthly). Each period was coded 1 (at least one report received) or 0 (no report received), with possible scores ranging from 0 (totally non-adherent) to 13 (totally adherent, 1 practice + 12 monthly TSSEs).

Demographic, cognitive and emotional factors were measured in a baseline questionnaire administered at the start of the ASICA trial.

Demographic factors.

Age in years and gender were self-reported. Socioeconomic status was coded 1 (most deprived) to 10 (least deprived) by linking postcodes to indices of deprivation (Scottish Index of Multiple Deprivation; English indices of deprivation (30,31). Rurality/Urbanity was coded from 1 (most urban) to 10 (most rural) (32,33).

Cognitive factors.

Intention towards engaging in TSSE was assessed with the item "In the next 12 months, do you intend to check your skin for early signs of skin cancer? [yes/no]…. If yes, how many times? [insert number]". In order to map intentions onto guideline behaviour, this item was scored categorically as <12 (intention below guideline), =12 (guideline intention) or >12 (intention above guideline).

Self-efficacy towards TSSE was assessed with 4 items rated on a 10-point scale from 'not at all confident' to 'highly confident': "How confident are you that……you can check your

For peer review only - http://bmjopen.bmj.com/site/about/guidelines.xhtml

own skin correctly?; that you will find time in the next 12 months to check your own skin?; that you will remember to check your own skin at least once a month?; that if you find a spot or mole of concern that you will take appropriate action?"

Action Planning about TSSE was assessed with 2 items rated on a 5-point scale from 'strongly agree to 'strongly disagree': "I have made plans about when to examine my own skin" and "I have made plans about where I will be when I examine my own skin".

All of the items measuring cognitive factors were developed for the ASICA study (to relate specifically to the TSSE context) but were based on standard and valid methods of assessing the relevant theoretical constructs (34, 35, 36).

Emotional factors.

Anxiety and depression were measured using the 14 item Hospital Anxiety & Depression Scale (HADS; 37), a valid measure with adequate internal consistency (38). Anxiety and depression were scored separately (from 0-4), with higher scores indicating greater distress.

Melanoma specific worries were assessed with the 4 item Melanoma Worry Scale (39). Items were rated on a 5 point scale from 'not at all' to 'almost all the time' and assessed the frequency and impact of worries about possible recurrence.

Analysis

Latent group mixture modelling (LGMM) was carried out with Mplus V8.4 (40) to establish if there were systematically different classes of TSSE over the 12 month measurement period. A series of models were estimated from the TSSE adherence data with 1 to 5 latent classes, and were compared on the Bayesian information criterion, entropy values and number of participants per class. Class membership was obtained solely on the basis of the adherence data (i.e., covariates were not included in the modelling exercise), and the resulting classification information was then combined with the other information to explore the differences between the classes using the classical three-step method (41). In the present analysis, the LGMM identified 3 classes of adherence behaviour (adherent, drop-off and non-adherent) by modelling the proportion of people who had completed the recommended monthly TSSE within each successive 30 day period over 12 months. Higher proportional estimates in this modelling exercise mean that more people are completing the recommended

For peer review only - http://bmjopen.bmj.com/site/about/guidelines.xhtml

TSSE within each time window. Logistic regressions were then conducted using SPSS v25 to predict membership of these 3 classes from demographic (age, gender, socioeconomic status, rurality), cognitive (intentions, self-efficacy and action plans about TSSE) and emotional (anxiety, depression and melanoma worry) factors. In these logistic regression analyses, the dependent variable was dummy coded (with the adherent coded as 0 and the non-adherent or drop-off coded as 1). Categorical intention scores were treated as unordered categories. Missing data, which were <4% on all measures other than intention and 11.5% for intention, were dealt with by item deletion. Intention analyses were re-run with missing values imputed and results did not change.

RESULTS

What does adherence to TSSE over 12 months look like? Are there distinct patterns of non-adherence?

Of the 104 participants, 12 did not submit any TSSE reports using ASICA and the remainder submitted between 1 and 13 (median 6.5, IQR 9.75).

LGMM was tested with 1 to 5 latent groups. The lowest (best) Bayesian Information Criterion (BIC) was for a 3 class solution (1318). The entropy score (a measure of the quality of the classification) for the 3 class solution was also highly satisfactory (.906). The number of participants per class was substantial with the best fitting model classifying reasonable numbers (n=25/n=36/n=43) into categories that were readily interpretable (as showing consistent non-adherence; adherence which dropped off over time; and consistent adherence respectively). The three class solution was therefore selected for further study.

Figure 1 shows the LGMM estimated and observed values plotted over time and illustrates the 3 distinct classes identified. The largest class are people who are adherent (n=43) who achieve over 90% adherence across the study period and show no significant decrease over time (p=.188). The next class, people whose adherence dropped-off over time (n=36), are those who start well, particularly after the first prompt but whose TSSE adherence declines steadily over 12 months (p< .001) until only around 20% are providing the required reports by the final month. The final group, people who are non-adherents (n=25) start poorly,

For peer review only - http://bmjopen.bmj.com/site/about/guidelines.xhtml

submitting only 28% of reports in the first month, and decrease rapidly such that they are providing virtually no reports by 6 months (p=.022). An overview of the characteristics of participants in the three TSSE adherence classes can be found in Table 1. The accuracy of the classification (likely versus actual classification) was high (adherent =0.98; drop-off =0.93; non-adherent = 0.96).

Table 1: Demographic, cognitive and emotional factors in the three adherence groups

	Adherent	Drop-Off	Non-Adherent
N	43	36	25
Age in years (M, SD)	60.45 (11.77)	57.12 (13.31)	58.50 (16.38)
Gender (M/F)	16/27	20/16	12/13
Rurality (urban/rural)	21/22	23/13	18/7
Social Deprivation (M, SD)	7.98 (1.50)	8.17 (1.72)	7.92 (2.14)
Action Planning (M, SD)	6.86 (2.10)	5.56 (2.47)	6.25 (2.42)
Self-Efficacy (M, SD)	32.32 (5.89)	28.85 (7.04)	30.50 (6.90)
Intentions (n)*			
<12 times	13	15	6
=12 times	22	11	7
>12 times	6	3	9
HADS Anxiety (Mean, SD)	4.23 (3.99)	5.33 (3.84)	6.13 (4.95)
HADS Depression (Mean, SD)	1.93 (2.19)	3.14 (3.45)	3.75 (3.05)
Melanoma Worry (Mean, SD)	8.33 (3.52)	8.73 (3.59)	8.75 (3.90)

* Only 92/104 participants completed the intention measure

Why does optimal and suboptimal TSSE adherence occur? That it is, do groups with suboptimal adherence differ from those with optimal adherence on demographic (age, gender, socioeconomic status, rurality) socio-cognitive (intentions, self-efficacy, action plans) and/or emotional (recurrence worries, anxiety, depression) factors?

The results of the univariate logistic regression analyses predicting adherence class (adherent; drop-off; non-adherent) from the demographic, cognitive and emotional measures are shown

For peer review only - http://bmjopen.bmj.com/site/about/guidelines.xhtml
For peer review only

in Table 2 (comparison of non-adherent and adherent) and Table 3 (comparison of drop-offs and adherent). Supplementary multivariate analyses can be found in Tables S1 and S2 in supplementary file S1.

Table 2: Logistic regression analyses of demographic, cognitive and emotional factors in people who are **non-adherent** (comparison group) **vs adherent.**

	B	SE	Wald	p	OR	95% CI OR
**Univariate**						
Age	0.01	0.02	0.33	0.566	0.99	[.95,1.03]
Gender	0.44	0.51	0.76	0.385	1.56	[.57,4.23]
SIMD	-0.02	0.14	0.02	0.9	0.98	[.74,1.30]
Rurality	-0.99	0.54	3.37	0.066	0.37	[.13,1.07]
Intention <12 **	-1.18	0.72	2.67	0.103	0.31	[.08,1.27]
Intention =12**	-1.55	0.68	5.17	0.023*	0.21	[.06,.81]
Action plan	-0.13	0.12	1.17	0.28	0.88	[.70,1.11]
Self-efficacy	-0.04	0.05	1.18	0.28	0.96	[.88,1.04]
HADS anxiety	0.1	0.06	2.76	0.096	1.10	[.98,1.24]
HADS depression	0.27	0.11	6.43	0.011*	1.31	[1.06,1.61]
MWS	0.04	0.07	0.23	0.633	1.04	[0.90,1.19]

* =significant at p<.05. ** Intention >12 used as comparison

Table 3: Logistic regression analyses of demographic, cognitive and emotional factors in people whose adherence **dropped-off** over time **(**comparison group) vs people who are adherent

	B	SE	Wald	p	OR	95% CI OR
**Univariate**						
Age	-0.02	0.02	1.38	0.241	0.98	[.94,1.02]
Gender	0.75	0.46	2.63	0.105	2.11	[.86,5.2]
SIMD	-0.08	0.14	0.28	0.597	1.08	[.81,1.43]
Rurality	-0.62	(0.46)	1.78	0.182	0.54	[.22,1.33]
Intention <12**	0.84	0.8	1.09	0.297	2.31	[.48,11.12]
Intention =12**	0	0.8	0	1	1	[.21,4.78]
Action plan	-0.25	0.11	5.77	0.016*	0.78	[.63,.96]
Self-efficacy	-0.08	0.04	4.82	0.028*	0.92	[.86,.99]

For peer review only - http://bmjopen.bmj.com/site/about/guidelines.xhtml

HADS Anxiety	0.07	0.06	1.52	0.217	1.08	[.96,1.21]
HADS Depression	0.16	0.09	3.19	0.074	1.17	[.98,1.40]
MWS	0.04	0.07	0.26	0.61	1.06	[.91,1.18]

* =significant at p<.05. ** Intention >12 used as comparison

Age, sex and SIMD did not significantly predict adherence class in any analysis, indicating that demographic factors alone are not sufficient to predict different adherence trajectories over time. However there was a tendency for rural participants to be more adherent.

In univariate comparisons of *cognitive factors*, people who were non-adherent were less likely to intend to carry out TSSE monthly than people who were adherent, instead reporting (potentially unrealistic) intentions to perform TSSE more often than monthly. People who were non–adherent did not differ from people who were adherent in terms of action plans or self-efficacy towards TSSE. People whose adherence dropped-off did not differ significantly from people who were adherent in their TSSE intentions. However the people whose adherence dropped-off reported lower levels of action planning and self-efficacy than people who were adherent. When *emotional factors* were compared, people who were non-adherent had significantly higher depression scores than people who were adherent with people whose adherence dropped off intermediate but not reliably different from people who were adherent. Anxiety was slightly higher in people who were non-adherent but this was not significant at p<.05. Melanoma worries did not significantly differ between any of the adherence groups.

DISCUSSION

Distinct patterns in TSSE adherence emerged in participants using the ASICA intervention over 12 months. Of the 104 participants, 41% (those in the 'adherent' category) consistently completed >90% of guideline recommended TSSEs over 12 months, which compares favourably to the 16-24% of participants reporting optimal TSSE adherence in a recent systematic review (18). However, even with the theory and evidence-based support offered by ASICA, the remaining 59% of participants in the present study showed one of two

For peer review only - http://bmjopen.bmj.com/site/about/guidelines.xhtml

patterns of suboptimal adherence. Approximately 35% of people (those in the 'drop-off' category) initially adhered well but showed steady and significant declines in TSSE adherence over time until only around a fifth were conducting TSSEs by 12 months. The remaining 24% (those in the 'non-adherent' category) consistently failed to engage in monthly TSSEs, with only a minority (around a third) conducting a TSSE in the first month and virtually none completing one from 6 months onwards.

Predictors of Adherence

One of the aims of the present study was to determine whether participants who would go on to display suboptimal adherence could be identified at baseline so that they could be better supported from intervention outset. To this end, adherence trajectories were predicted from a range of easily measurable and theoretically relevant demographic, cognitive and emotional factors. None of the demographic factors assessed in the current study (age, gender, socioeconomic status, rurality) differentiated between classes of TSSE adherence behaviour, although there was a slight tendency for those living in urban areas to be less adherent. It should be noted however, that participants in this study showed extremely limited variance in socioeconomic status, tending to be more affluent than the general population. This restricted range likely reflects the combined effects of melanoma incidence being higher in more affluent individuals (e.g., 42), and trial participation being skewed towards individuals from higher socioeconomic groups, and limits the conclusions that can be drawn about socioeconomic status.

When cognitive factors (intentions about TSSE, self-efficacy towards TSSE and plans about when and where to perform TSSE) were examined, the data suggested that different cognitive processes may be involved in the two suboptimal patterns of adherence observed ('non-adherent' and 'drop-off'). People who were non-adherent were significantly more likely to have high and potentially unrealistic intentions about TSSE than people who were adherent: with many intending to perform TSSEs more frequently than monthly. Neither TSSE self-efficacy nor action planning significantly differentiated people who were non-adherent from people who were adherent. In contrast, the opposite pattern was found to differentiate the people whose adherence dropped off from people who were adherent: the two groups did not significantly differ in their intentions about TSSE but people whose adherence dropped-off showed significantly lower levels of action planning and self-efficacy than people who were adherent. The observed pattern of results fits well with theoretical models of health behaviour change such as the Health Action Process Approach (HAPA; 43).

For peer review only - http://bmjopen.bmj.com/site/about/guidelines.xhtml

HAPA explicitly distinguishes between motivational processes (such as intentions) involved in the early goal-setting phase of behaviour change, and volitional processes (such as self-efficacy and planning) involved in the later goal-pursuit phase of behaviour change. In the present study, the participants who were least likely to 'get started' (i.e., the people who were non-adherent) showed measurable differences in motivational processes (intentions) relative to people who were adherent. Similarly, those who started well (had motivation) but then had issues with maintaining the volitional aspects of regular TSSE over time (i.e., those whose adherence dropped-off) showed lower levels of self-efficacy and planning than those who were adherent. Cognitive factors were on average less predictive of TSSE behaviour in the present study than in other melanoma studies (22), possibly because the ASICA intervention was designed to boost intentions, self-efficacy and planning. This may have reduced natural variance in these constructs, limiting their predictive power.

The analysis of the emotional factors (anxiety, depression and melanoma specific worries) indicated that while people who were adherent consistently had the 'best' scores (i.e., lowest anxiety, lowest depression, fewest worries about recurrence), the only factor that significantly differentiated peoples who were adherent from people who were non-adherent was depression. People whose adherence dropped-off in contrast, did not significantly differ from people who were adherent on any of the emotional factors. It is noteworthy that melanoma specific worries were remarkably consistent across adherence groups and did not predict TSSE behaviour. Fears about recurrence are often suggested as a likely predictor of TSSE behaviour (i.e., that people worried about recurrence will check their skin more frequently, potentially more frequently than recommended), and recent evidence suggests that people who are more worried about recurrence are more likely to conduct TSSE and to conduct more thorough TSSE (44). The present results were not in line with this, suggesting that recurrence fears were unrelated to TSSE adherence over 12 months.

For peer review only

Clinical Implications

The present results suggest that routinely screening for fear of recurrence will not helpfully discriminate melanoma patients in terms of their likely future TSSE adherence. Making baseline assessments of participants' intentions about TSSE and level of depression however may serve to highlight the group most likely to be non-adherent. Intentions, if suboptimal, can in theory be strengthened with three behaviour change techniques: giving information about the health consequences of TSSE, setting goals about completing TSSE and providing an incentive for reaching the outcome (45). Similarly, depression might be reduced by

For peer review only - http://bmjopen.bmj.com/site/about/guidelines.xhtml

helping patients to more effectively manage negative emotions, e.g., through stress management techniques (45). It is important to note however that the effect sizes in the present study were small so the potential clinical benefit of this approach would be limited.

Identifying those who start well but falter (those whose adherence 'drops-off') is challenging. While measures of self-efficacy and action planning differentiated people whose adherence dropped off from people who were adherent on average, they would not reliably identify people on this adherence trajectory at baseline (e.g., baseline self-efficacy scores in the present data set would correctly identify only 46% of drop-offs). Instead, adherence behaviour would have to be monitored for 6 months (in the present data), to identify 75% of people whose adherence dropped off (as those who have completed <4 of the recommended 6 TSSEs). Top up interventions could be targeted at this point: action planning might be addressed by prompting people to make 'if-then' plans (23) and self-efficacy might be enhanced by methods such as verbal persuasion and focusing on past success (e.g., in adhering in the early stages) (45). If top-up interventions were to be used, it would be possible in the present data to accurately identify 92% of people who were non-adherent and 100% of people who were adherent at 3 months on the basis of TSSEs completed (0-1 vs 2-3).

Strengths and Limitations

The present study has several methodological strengths. It uses objectively recorded TSSE behaviour rather than self-reports, and we can therefore be confident that TSSE took place at the times indicated. The study also followed participants over a full 12 months and tested both emotional and socio-cognitive models of behaviour. However, as discussed above, one notable limitation of the current study is that participants came almost exclusively from the less deprived end of the socioeconomic spectrum. While broadly representative of the clinical population (melanoma being more common in high SES individuals) the results reported are not representative of the general population. This raises an important point about whether digital interventions like ASICA would exacerbate existing health inequalities if uptake (and therefore potential to benefit) is higher in individuals from less deprived sectors of the population (cf.46,47), or whether they would usefully 'free up' face to face resources for those least likely to engage with remote healthcare

Future Directions

For peer review only - http://bmjopen.bmj.com/site/about/guidelines.xhtml

Future studies should investigate in more detail methods for early detection of those least likely to be adherent. Based on our data, interventions which boost action planning and self-efficacy at 3 and 6 months might be beneficial in reducing the number people whose adherence drops-off while an intervention prior to starting monthly TSSE to address motivation and depression might reduce the number of people who go on to be non-adherent. Detection of sub-optimal adherence on an individual level would require real time measurement and more dynamic and individualised assessment in order to model the thresholds beyond which optimal adherence can be reliably distinguished from adherence which is likely to decline in future. Future studies should also extend the present work on the frequency and maintenance of TSSE to TSSE thoroughness. Studies which have investigated the thoroughness of TSSE, suggest that only 7-14% of patients check their whole body while performing TSSE (44,48) and only 9-13% use a mirror or ask another person to help them check areas that are difficult to see (48). Melanoma can recur anywhere on the body and more thorough SSE is associated with detection of earlier stage, more survivable lesions (49).

In conclusion, the present study demonstrates that adherence to recommended monthly TSSE in individuals treated for melanoma can be reliably differentiated into three distinct trajectories over time: adherent, drop-off and non-adherent. Collecting information about intentions to engage in TSSE, depression level, self-efficacy and/or action planning at outset may help to distinguish between those who go on to engage in optimal, declining or consistently suboptimal adherence.

Author contributions

JA, MJ, DWJ and PM conceived of, designed and planned the study. DWJ analysed the data in consultation with JA, MJ and PM. JA drafted the manuscript. JA, DWJ, MJ and PM interpreted the results and revised the manuscript. JA is responsible for the overall content.

Funding statement

This work was supported by a grant from a Cancer Research UK Population Research Committee project award (C10673/A21685). The views and opinions expressed herein are those of the authors and do not necessarily reflect those of Cancer Research UK. The funder (through their peer review and funding board review process) approved the study proposal but had no role in the collection, analysis, or interpretation of data, or writing of the report.

For peer review only - http://bmjopen.bmj.com/site/about/guidelines.xhtml

Competing interests

None of the authors have competing interests to declare.

Ethics Approval

The project received full approval from the North of Scotland Research Ethics Committee on 28th April 2017 (17/NS/0040). Written informed consent was obtained from all study participants

Data sharing

Data are available on request.

Figure Legend

Figure 1: The 3 classes of adherence to TSSE over time showing LGMM estimated (statistically predicted) and observed (actual) proportion of participants completing TSSE within each 30 day period.

References

1. Cancer Research UK. Melanoma skin cancer statistics 2015-17. Retrieved from https://www.cancerresearchuk.org/health-professional/cancer-statistics/statistics-by-cancer-type/melanoma-skin-cancer

2. Francken AB, Accortt NA, Shaw HM, *et al*. Follow-up schedules after treatment for malignant melanoma. *Br J Surg* 2008;95:1401-1407. https://doi.org/10.1002/bjs.6347

3. Di Fronzo LA, Wanek LA, Elashoff R *et al*. Increased incidence of second primary melanoma in patients with a previous cutaneous melanoma. *Ann Surg Oncol* 1999;6:705711.

4. National Institute for Health and Care Excellence (NICE). Melanoma: Assessment and management (NG14; 2015). Retrieved from https://www.nice.org.uk/guidance/ng14

5. Health Improvement Scotland. SIGN QRG 146 Cutaneous Melanoma. 2016. Retrieved from https://www.sign.ac.uk/assets/qrg146.pdf

6. Berwick M, Begg CB, Fine JA. Screening for cutaneous melanoma by skin self-examination. *J Natl Cancer Inst* 1996;88:17–23.

7. Watts CG, Dieng M, Morton RL, *et al*. Clinical practice guidelines for identification, screening and follow-up of individuals at high risk of primary cutaneous melanoma: a systematic review. *Br J Dermatol* 2015;172:33–47. https://doi.org/10.1111/bjd.13403.

For peer review only - http://bmjopen.bmj.com/site/about/guidelines.xhtml

8. Dicker TJ, Kavanagh GM, Herd RM, *et al*. A rational approach to melanoma follow-up in patients with primary cutaneous melanoma. Scottish Melanoma Group. *Br J Dermatol* 1999;140:249–254. https://doi.org/10.1046/j.1365-2133.1999.02657.x

9. Meyers MO, Yeh JJ, Frank J, *et al* (2009). Method of detection of initial recurrence of stage II/III cutaneous melanoma: analysis of the utility of follow-up staging. *Ann Surg Oncol*, 2009;16:941–947. https://doi.org/10.1245/s10434-008-0238-y

10. Auckland R, Wassell P, Hall S, *et al.* Exploring patterns of recurrent melanoma in Northeast Scotland to inform the introduction a digital self-examination intervention. *BMC Dermatol* 2014;14:4. https://doi.org/10.1186/1471-5945-14-4

11. Brady MS, Oliveria SA, Christos PJ, *et al*. Patterns of detection in patients with cutaneous melanoma. *Cancer* 2000;89:342–347.

12. Swetter SM, Pollitt RA, Johnson TM, *et al*. Behavioral determinants of successful early melanoma detection: role of self and physician skin examination. *Cancer* 2012;118:3725–34. https://doi.org/10.1002/cncr.26707.

13. Moore Dalal K, Zhou Q, Panageas KS, *et al*. Methods of detection of first recurrence in patients with stage I/II primary cutaneous melanoma after sentinel lymph node biopsy. *Ann Surg Oncol* 2008;15:2206-2214. doi: 10.1245/s10434-008-9985-z

14. Paddock LE, Lu SE, Bandera EV, *et al*. Skin self-examination and long-term melanoma survival. *Melanoma Res* 2016;26:401–408.

15. Hull PR, Piemontesi NG, Lichtenwald J. Compliance with self-examination surveillance in patients with melanoma and atypical moles: an anonymous questionnaire study. *J Cutan Med and Surg* 2011;15:97–102. https://doi.org/10.2310/7750.2011.10011

16. Körner A, Coroiu A, Martins C, *et al*. Predictors of skin self-examination before and after a melanoma diagnosis: the role of medical advice and patient's level of education. *Int Arch Med* 2013;6:8. https://doi.org/10.1186/1755-7682-6-8

17. Manne S, Lessin S. Prevalence and Correlates of Sun Protection and Skin Self-Examination Practices Among Cutaneous Malignant Melanoma Survivors. *J Behav Med* 2006;29**:**419–434. https://doi.org/10.1007/s10865-006-9064-5

18. Bhurosy T, Heckman CJ, Riley M. Prevalence and correlates of skin self-examination behaviors among melanoma survivors: a systematic review, *Transl Behav Med* 2020;10:1120–1133, https://doi.org/10.1093/tbm/ibaa003

19. Weinstock MA, Risica PM, Martin RA, *et al*. Melanoma early detection with thorough skin self-examination: the "Check It Out" randomized trial. *Am J Prev Med* 2007; 32:517–524. https://doi.org/10.1016/j.amepre.2007.02.024

For peer review only - http://bmjopen.bmj.com/site/about/guidelines.xhtml

20. Janda M, Neale RE, Youl P, *et al*. Impact of a video-based intervention to improve the prevalence of skin self-examination in men 50 years or older: the randomized skin awareness trial. *Arch Dermatol* 2011;147:799–806. https://doi.org/10.1001

21. Coroiu A, Moran C, Bergeron C, *et al.* Short and long-term barriers and facilitators of skin self-examination among individuals diagnosed with melanoma. *BMC Cancer* 20**;**123. https://doi.org/10.1186/s12885-019-6476-5

22. Coroiu A, Moran C, Bergeron C, *et al* (2020a). Operationalization of skin self-examination in randomized controlled trials with individuals at increased risk for melanoma: A systematic review. *Pat Ed Couns*, 2020;*103*:1013–1026. https://doi.org/10.1016/j.pec.2019.12.009

23. Gollwitzer PM. Implementation intentions: strong effects of simple plans. *Am Psychol* 1999;54:493.

24. Murchie P, Allan JL, Brant W, *et al*. Total skin self-examination at home for people treated for cutaneous melanoma: development and pilot of a digital intervention. *BMJ Open* 2015;5:e007993

25. Murchie P, Masthoff J, Walter FM, *et al.* Achieving Self-Directed Integrated Cancer Aftercare (ASICA) in melanoma: protocol for a randomised patient-focused pilot trial of delivering the ASICA intervention as a means to earlier detection of recurrent and second primary melanoma. *Trials* 2019;20**:**318. https://doi.org/10.1186/s13063-019-3453-x

26. Fisher JD, Fisher WA. Changing AIDS-risk behavior. *Psychol Bull* 1992;111:455–74

27. Carver CS, Scheier MF. Attention and self-regulation: a control theory approach to human behaviour. New York: Springer; 1981.

28. Murchie P, Constable L, Hall S, *et al*. A randomised patient-focused trial of the Achieving Self-Directed Integrated Cancer Aftercare (ASICA) intervention for detection of recurrent and second primary melanoma: effect on psychological well-being and quality of life. In submission.

29. Keung EZ, Gershenwald JE. The eighth edition American Joint Committee on Cancer (AJCC) melanoma staging system: implications for melanoma treatment and care. *Expert Rev Anticancer Ther*, 2018;18:775–784. https://doi.org/10.1080/14737140.2018.1489246

30. Scottish Government. Scottish Index of Multiple Deprivation. 2020. Retrieved from https://www.gov.scot/collections/scottish-index-of-multiple-deprivation-2020/

31. UK Government. National Statistics English indices of deprivation 2019. Retrieved from https://www.gov.uk/government/statistics/english-indices-of-deprivation-2019

32. Scottish Government. Urban Rural Classification 2016. Retrieved from https://www.gov.scot/publications/scottish-government-urban-rural-classification-2016/pages/2/

For peer review only - http://bmjopen.bmj.com/site/about/guidelines.xhtml

33. Office for National Statistics. Rural/urban classification 2011. Retrieved from https://www.ons.gov.uk/methodology/geography/geographicalproducts/ruralurbanclassifications

34. Francis J, Eccles MP, Johnston M, Walker AE, Grimshaw JM, Foy R, Kaner EFS, Smith L, Bonetti D. Constructing questionnaires based on the theory of planned behaviour: A manual for health services researchers. Newcastle upon Tyne, UK 2004: Centre for Health Services Research, University of Newcastle upon Tyne.

35. Burrell AMG, Allan JL, Williams DM, Johnston M. What do self-efficacy items measure? Examining the discriminant content validity of self-efficacy items. *Br J Health Psychol*, 2018;23:597-611.

36. Sniehotta FF, Scholz U, Schwarzer R. Bridging the intention–behaviour gap: Planning, self-efficacy, and action control in the adoption and maintenance of physical exercise, *Psychol Health* 2005;20:143-160

37. Zigmond AS, Snaith RP. The hospital anxiety and depression scale. *Acta Psychiat Scand* 1983;67:361-70.

38. Bjelland I, Dahl AA, Haug TT, et al. The validity of the Hospital Anxiety and Depression Scale: An updated literature review. *J Psychosom Res* 2002;52:69-77.

39. Moye MS, King SMC, Rice ZP, *et al*. Effects of total-body digital photography on cancer worry in patients with atypical mole syndrome. *JAMA Dermatol*, 2015;151:137–43. https://doi.org/10.1001/jamadermatol.2014.2229

40. Muthen LK, Muthen BO. Mplus User Guide. Eighth Edition. 1988-2017. Los Angeles, CA: Muthen & Muthen.

41. Wang J, Wang X. Structural Equation Modeling: Applications Using Mplus. Second Edition. 2020. John Wiley & Sons.

42. Johnson-Obaseki SE, Labajian V, Corsten MJ, *et al*. Incidence of cutaneous malignant melanoma by socioeconomic status in Canada: 1992-2006. *J Otolaryng Head Neck Surg* 2015;*44*:53. https://doi.org/10.1186/s40463-015-0107-1

43. Schwarzer R. Self-efficacy in the adoption and maintenance of health behaviours: Theoretical approaches and a new model. In: Schwarzer R, editor. Self-Efficacy: Thought Control of Action. Washington: Taylor and Francis; 1992. pp. 217–243.

44. Manne SL, Heckman CJ, Kashy D, *et al*. Prevalence and correlates of skin self-examination practices among cutaneous malignant melanoma survivors. *Prev Med Rep* 2020;19. https://doi.org/10.1016/j.pmedr.2020.101110.

45. Johnston M, Carey RN, Connell LE, *et al*. Development of an online tool for linking behavior change techniques and mechanisms of action based on triangulation of findings from literature synthesis and expert consensus. *Translat Behav Med* 2021;5:1049-1065. https://doi.org/10.1093/tbm/ibaa050

For peer review only

46. McAuley A. Digital health interventions: widening access or widening inequalities? *Pub Health* 2014;12:1118-1120.

47. Honeyman M, Maguire D, Evans H, *et al*. Digital technology and health inequalities: a scoping review. 2020. Cardiff: Public Health Wales NHS Trust.

48. Coups EJ, Manne SL, Stapleton JL, *et al*. Skin self-examination behaviors among individuals diagnosed with melanoma. *Melanoma Res* 2016;26:71–6. https://doi.org/10.1097/CMR.0000000000000204.

49. Pollitt RA, Geller AC, Brooks DR, *et al*. Efficacy of skin self-examination practices for early melanoma detection. *Cancer Epidemiol Biomark Prev* 2009;18:3018–23. https://doi.org/10.1158/1055-9965.epi-09-0310.

For peer review only

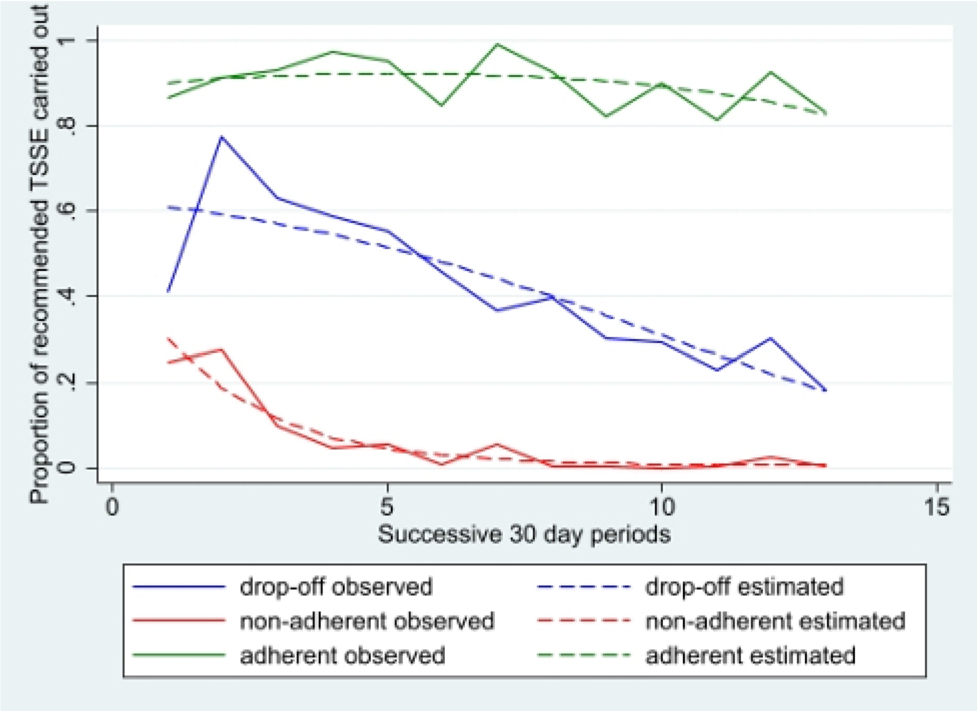

Figure 1: The 3 classes of adherence to TSSE over time showing LGMM estimated and observed values

172x125mm (72 x 72 DPI)

Supplementary File S1

Multivariate analyses

The main analyses reported in the manuscript are univariate logistic regressions. As a supplementary analysis, any predictors that were significant at p<.1 were entered into further multivariate analyses. These multivariate analyses are shown below in Tables S1 and S2.

When intention, anxiety and depression (i.e. all predictors where p<.1) were included in the analysis of non-adherent versus adherent participants, intentions about TSSE and depression remained significant, each explaining unique variance. People who were non-adherent were more depressed and more likely to intend to conduct TSSE more frequently than recommended than people who were adherent.

When action planning and self-efficacy (as predictors where p<.1) were included in a multivariate analysis of the drop offs versus the adherent participants, neither was significant. This may reflect the fact that efficacy is a theoretical precursor of action planning, and as such, the two will be highly inter-related and may explain the same variance.

Table S1: Multivariate logistic regression analyses of demographic, cognitive and emotional factors in people who are **non-adherent** (comparison group) **vs adherent.**

	B	SE	Wald	p	OR	95% CI OR
**Multivariate**						
Rurality	-0.91	0.64	2.05	0.152	0.40	[.12,1.40]
Intention<12	-1.69	0.84	4.09	0.043*	0.18	[.04,.95]
Intention=12	-1.70	0.74	5.31	0.021*	0.18	[.04,.78]
HADS Anxiety	-0.01	0.09	0.03	0.87	0.99	[.83,1.17]
HADS Depression	0.34	0.15	4.91	0.027*	1.40	[1.04,1.89]

* =significant at p<.05. ** Intention >12 used as comparison

For peer review only - http://bmjopen.bmj.com/site/about/guidelines.xhtml

Table S2: Multivariate logistic regression analyses of demographic, cognitive and emotional factors in people whose adherence **dropped-off** over time **(**comparison group) vs people who are adherent

	B	SE	Wald	p	OR	95% CI OR
**Multivariate**						
Action Plan	-0.14	0.13	1.13	0.289	0.87	[.68,1.12]
Self-efficacy	-0.05	0.05	1.47	0.225	0.95	[.87,1.03]
HADS depression	0.14	0.09	2.28	0.132	1.15	[.96,1.38]

* =significant at p<.05. ** Intention >12 used as comparison

For peer review only

STROBE 2007 (v4) checklist of items to be included in reports of observational studies in epidemiology*
Checklist for cohort, case-control, and cross-sectional studies (combined)

Section/Topic	Item #	Recommendation	Reported on page #
**Title and abstract**	1	(*a*) Indicate the study's design with a commonly used term in the title or the abstract	p1-2
		(*b*) Provide in the abstract an informative and balanced summary of what was done and what was found	p2
**Introduction**			
Background/rationale	2	Explain the scientific background and rationale for the investigation being reported	p4-5
Objectives	3	State specific objectives, including any pre-specified hypotheses	p5
**Methods**			
Study design	4	Present key elements of study design early in the paper	p5-6
Setting	5	Describe the setting, locations, and relevant dates, including periods of recruitment, exposure, follow-up, and data collection	p6-7
Participants	6	(*a*) *Cohort study*—Give the eligibility criteria, and the sources and methods of selection of participants. Describe methods of follow-up<br>*Case-control study*—Give the eligibility criteria, and the sources and methods of case ascertainment and control selection. Give the rationale for the choice of cases and controls<br>*Cross-sectional study*—Give the eligibility criteria, and the sources and methods of selection of participants	p7
		(*b*) *Cohort study*—For matched studies, give matching criteria and number of exposed and unexposed<br>*Case-control study*—For matched studies, give matching criteria and the number of controls per case	N/A
Variables	7	Clearly define all outcomes, exposures, predictors, potential confounders, and effect modifiers. Give diagnostic criteria, if applicable	p7-8
Data sources/ measurement	8*	For each variable of interest, give sources of data and details of methods of assessment (measurement). Describe comparability of assessment methods if there is more than one group	p7-8
Bias	9	Describe any efforts to address potential sources of bias	p7-9
Study size	10	Explain how the study size was arrived at	p7
Quantitative variables	11	Explain how quantitative variables were handled in the analyses. If applicable, describe which groupings were chosen and why	p8-9
Statistical methods	12	(*a*) Describe all statistical methods, including those used to control for confounding	p8-9
		(*b*) Describe any methods used to examine subgroups and interactions	p8-9
		(*c*) Explain how missing data were addressed	p9
		(*d*) *Cohort study*—If applicable, explain how loss to follow-up was addressed<br>*Case-control study*—If applicable, explain how matching of cases and controls was addressed	N/A

For peer review only

		Cross-sectional study—If applicable, describe analytical methods taking account of sampling strategy	
		(*e*) Describe any sensitivity analyses	p9
**Results**			
Participants	13*	(a) Report numbers of individuals at each stage of study—eg numbers potentially eligible, examined for eligibility, confirmed eligible, included in the study, completing follow-up, and analysed	Table 1
		(b) Give reasons for non-participation at each stage	N/A
		(c) Consider use of a flow diagram	N/A
Descriptive data	14*	(a) Give characteristics of study participants (eg demographic, clinical, social) and information on exposures and potential confounders	Table 1
		(b) Indicate number of participants with missing data for each variable of interest	p9, Table 1
		(c) *Cohort study*—Summarise follow-up time (eg, average and total amount)	p7
Outcome data	15*	*Cohort study*—Report numbers of outcome events or summary measures over time	p9
		Case-control study—Report numbers in each exposure category, or summary measures of exposure	
		Cross-sectional study—Report numbers of outcome events or summary measures	
Main results	16	(*a*) Give unadjusted estimates and, if applicable, confounder-adjusted estimates and their precision (eg, 95% confidence interval). Make clear which confounders were adjusted for and why they were included	Tables 2 and 3
		(*b*) Report category boundaries when continuous variables were categorized	p8-9
		(*c*) If relevant, consider translating estimates of relative risk into absolute risk for a meaningful time period	N/A
Other analyses	17	Report other analyses done—eg analyses of subgroups and interactions, and sensitivity analyses	p7
**Discussion**			
Key results	18	Summarise key results with reference to study objectives	p9-12
Limitations	19	Discuss limitations of the study, taking into account sources of potential bias or imprecision. Discuss both direction and magnitude of any potential bias	p15-16
Interpretation	20	Give a cautious overall interpretation of results considering objectives, limitations, multiplicity of analyses, results from similar studies, and other relevant evidence	p13-15
Generalisability	21	Discuss the generalisability (external validity) of the study results	p15-16
**Other information**			
Funding	22	Give the source of funding and the role of the funders for the present study and, if applicable, for the original study on which the present article is based	p16

*Give information separately for cases and controls in case-control studies and, if applicable, for exposed and unexposed groups in cohort and cross-sectional studies.

Note: An Explanation and Elaboration article discusses each checklist item and gives methodological background and published examples of transparent reporting. The STROBE checklist is best used in conjunction with this article (freely available on the Web sites of PLoS Medicine at http://www.plosmedicine.org/, Annals of Internal Medicine at http://www.annals.org/, and Epidemiology at http://www.epidem.com/). Information on the STROBE Initiative is available at www.strobe-statement.org.

For peer review only
